# The modal-modular model of animal self-representation: a comparative and operational framework

**DOI:** 10.3389/fpsyg.2026.1885694

**Published:** 2026-06-25

**Authors:** Ivan A. Khvatov

**Affiliations:** Moscow Institute of Psychoanalysis, Moscow, Russia

**Keywords:** animal cognition, animal self-representation, body awareness, comparative psychology, modularity, operational framework, self-awareness

## Abstract

Research on animal self-awareness has long been organized around the mirror test and related self-recognition paradigms, encouraging a binary framing of whether a given species does or does not possess self-awareness. However, accumulating evidence suggests that animal self-representation cannot be reduced to a single experimental criterion, sensory modality, or linear evolutionary scale. This article proposes a comparative and operational framework for analyzing animal self-representation as a modal-modular regulatory architecture through which an organism takes its own body, actions, and agency into account in behavioral regulation. First, we clarify the distinctions among self-processing, self-representation, self-awareness, and self-consciousness, and distinguish three levels of self-representation: implicit bodily self-representation, minimal self-related awareness as anticipatory bodily and agentive regulation, and reflective self-representation, or self-consciousness. Second, we introduce the Modal-Modular Model, in which self-representation is analyzed along three functional dimensions: sensory input, represented bodily or agentive parameter, and motivational-behavioral context. Within this framework, we specify several candidate modules, including body size/passability, body weight/support, agency/action control, appearance-related self-representation, and modality-specific self-signal recognition. Third, we outline principles for operationalizing and validating these modules by matching tasks to species-specific sensory ecology, identifying target bodily and agentive parameters, separating epistemic from pragmatic actions, distinguishing module-specific evidence from alternative explanations, and using behavioral contrasts, dependent variables, transfer tests, control conditions, evidence grades, and falsification routes. The proposed framework shifts the focus from asking whether animals “have” self-awareness as a unitary capacity to constructing species-specific profiles of self-representation across modules, sensory channels, ecological contexts, and operational criteria.

## 1 Introduction

The experimental study of self-awareness in animals is commonly traced back to Gallup’s seminal work (Gallup, 1970), in which mirror self-recognition (MSR) was introduced as one of the first operational criteria for studying self-recognition in non-human animals. This paradigm moved the topic from largely philosophical debate into experimental comparative psychology and stimulated a broad body of research across taxa (Gallup, 1982; Anderson and Gallup, 2011; Suddendorf and Butler, 2013; Gallup and Anderson, 2020; Priestley and Wascher, 2026). At the same time, the heuristic success of MSR encouraged a specific paradigm of visual self-recognition to function almost as a general criterion for self-awareness, thereby placing the question of animal selfhood within a binary pass/fail logic (de Waal, 2019; Gallup and Anderson, 2020; Lage et al., 2022).

Over the past decades, a substantial body of methodological and conceptual criticism has accumulated around MSR, showing the limits of this paradigm as a general framework for studying animal self-representation. The main difficulties can be summarized in five points:

1. Sensory bias: MSR relies on vision and mirror-based cues, which limits its applicability to species for which olfactory, tactile, vibrissal, hydrodynamic, or other non-visual channels are more behaviorally relevant (Bekoff, 2001; Krupa et al., 2001; Coombs and Netten, 2005; Chambers et al., 2014; Holzman et al., 2014; Horowitz, 2017; Mogdans, 2019; Yokose et al., 2024; Khvatov, 2025).
2. Morphological constraints: Successful performance in the mirror test often requires the ability to act on the marked body part, which is unavailable to many species and body organizations (Plotnik et al., 2006; de Waal, 2019; Kohda et al., 2019, 2022; Loth et al., 2022).
3. Motivational, social, and action-level ambiguity: Establishing a relation between the reflection and one’s own body may remain at the level of exploration, avoidance, social response, or epistemic engagement without transition to mark-directed pragmatic action (Fox, 1982; Anderson, 1984; Asendorpf et al., 1996; Shillito et al., 1999; Posada and Colell, 2007; Kopp et al., 2021; Murray et al., 2022; Vanhooland et al., 2025).
4. Ecological invalidity: Vertical reflective surfaces are rarely encountered in natural environments, making the testing situation biologically atypical for many species (Clary and Kelly, 2016; Calmette and Meunier, 2023; Lei, 2023; Priestley and Wascher, 2026).
5. Interpretative reductionism: MSR captures a specific form of visual self-representation, whereas its outcomes are often used to draw conclusions about self-awareness as a whole (Suddendorf and Butler, 2013; de Waal, 2019; Birch et al., 2020; Gallup and Anderson, 2020; Lage et al., 2022; Priestley and Wascher, 2026).

In response to these limitations, comparative psychology has increasingly developed gradualist, multi-component, embodied, and ecologically oriented approaches to self-awareness and self-representation (de Waal, 2019; Birch et al., 2020; Lage et al., 2022; Laurenzi et al., 2025). From this perspective, different aspects of self-representation may be expressed unevenly, depending on the animal’s morphology, sensory ecology, and behavioral problems. This orientation is already reflected in paradigms that move beyond the classical mirror test, including olfactory “mirror” tests, weight-awareness tasks, and passability judgments based on body size (Dale and Plotnik, 2017; Horowitz, 2017; Khvatov et al., 2023, 2024; Dobos and Pongrácz, 2025).

Despite substantial progress in the comparative study of self-representation, three key issues remain unresolved:

1. Conceptual fragmentation: terms such as self-awareness, self-recognition, self-representation, body awareness, minimal self, and self-consciousness are often used in overlapping ways. This leads to a conflation of levels of analysis and to situations in which distinct experimental paradigms are treated as addressing the same phenomenon, although they target different aspects of self-representation (Gallagher, 2000; Rochat, 2003; Morin, 2006; Suddendorf and Butler, 2013; Lage et al., 2022; Laurenzi et al., 2025).
2. Insufficient specification of modular structure: the multi-component nature of self-representation is increasingly acknowledged, yet a systematic framework is still needed to delineate candidate modules, their functional roles, their sensorimotor grounding, and the conditions under which they become behaviorally expressed (Povinelli and Cant, 1995; Bekoff and Sherman, 2004; Lenkei et al., 2020; Lage et al., 2022; Pongrácz et al., 2023; Dobos and Pongrácz, 2025; Laurenzi et al., 2025).
3. A gap between theoretical models and operationalization: existing multidimensional approaches, including the pattern theory of self and related frameworks, provide valuable conceptual maps, while comparative psychology still lacks an operational matrix linking self-related parameters, sensory modalities, ecological tasks, behavioral contrasts, evidence levels, controls, validation routes, and species-specific profile construction (Gallagher, 2013; Birch, 2022; Crump and Birch, 2022; Laurenzi et al., 2025; Priestley and Wascher, 2026).

Accordingly, the central challenge for comparative psychology is to identify which components of self-representation are expressed in a given species, through which sensorimotor modalities they become accessible, and under which ecologically valid conditions they can be empirically investigated. This article proposes the Modal-Modular Model of animal self-representation (MMM) as a profile-based and operational architecture for differentiating, comparing, and experimentally testing these components across species-specific ecological, morphological, and behavioral contexts. To this end, the article first clarifies the distinctions among self-processing, self-representation, self-awareness, and self-consciousness and outlines three levels of self-representation; it then develops the MMM in terms of functional dimensions and candidate modules, including body size/passability, body weight/support, agency/action control, appearance-related, and modality-specific self-signal recognition domains; finally, it formulates principles for operationalization and validation by linking theoretical distinctions to experimental tasks, dependent variables, behavioral criteria, evidence levels, control conditions, and alternative explanations. Supplementary Table 1 provides an extended operational matrix for applying this logic to candidate modules of animal self-representation.

## 2 What do we mean by “self”? Conceptual disambiguation for comparative research

In comparative research on animal self-representation, methodological limitations of particular experimental paradigms are compounded by the terminological heterogeneity of the field. Terms such as self-awareness, self-recognition, self-representation, body awareness, minimal self, self-consciousness, body schema, and body image are often used in partially overlapping ways, designating different levels of self-related organization or closely related phenomena described in different vocabularies (Head and Holmes, 1911; Gallup, 1970; Gallagher, 2000, 2013; Rochat, 2003; Morin, 2006; de Vignemont, 2010; Smith, 2017; Birch et al., 2020; Lage et al., 2022; Pongrácz et al., 2023; Laurenzi et al., 2025). In comparative psychology, this heterogeneity is especially consequential because, in the absence of verbal reports, paradigms such as mirror and olfactory self-recognition, body-as-obstacle and passability tasks, agency paradigms, metacognitive uncertainty tasks, or theory-of-mind-related paradigms may be overinterpreted as evidence for or against self-awareness as a whole, although they target different components of self-related organization.

The present section introduces a working conceptual vocabulary for relating terms, levels of organization, and experimental criteria in comparative animal research, without aiming to provide a full philosophical theory of the self or subjectivity. In this article, self-representation is used as the broadest category within the domain of self-related regulatory organization, but not as a synonym for adaptive sensorimotor regulation in general. The term refers to cases in which an organism’s own bodily or agentive parameters function as variables in the regulation of action; such parameters may include, for example, body size, body weight, bodily boundaries, action authorship, controllability, own signals, and the sensory consequences of self-produced action. This restriction is necessary because many adaptive behaviors can be explained by sensorimotor calibration, affordance perception, reinforcement history, or learned motor routines without requiring a self-representational interpretation. Self-consciousness, accordingly, is treated as the reflective form of self-representation, in which the self becomes an object of epistemic, symbolic, communicative, and socially mediated relation. In what follows, we distinguish three levels of self-related organization: implicit bodily self-representation, minimal self-related awareness, and reflective self-representation, or self-consciousness.

### 2.1 Implicit bodily self-representation: self-processing, body schema, and action-centered regulation

At the most basic level, self-representation can be understood as the organization of self-related information through which an organism takes its own body and actions into account during behavior. In this sense, self-processing refers to the ongoing processing of information about bodily position, movement, bodily boundaries, the sensory consequences of self-generated actions, and the relation between the body and the environment. Self-representation, in a stricter sense, refers to the relatively stable but continuously updated organization of such information into action-guiding models in which the organism’s own bodily or agentive parameters become behaviorally relevant variables. These two aspects form an interdependent process: ongoing processing maintains self-representation, while self-representation structures subsequent processing (Cummins, 1983; Machamer et al., 2000). Applied to animals, this provides a broad but constrained understanding of self-representation, requiring neither reflection, verbal report, nor conscious knowledge of oneself, while avoiding the equation of self-representation with adaptive sensorimotor regulation as such.

The classical notion most closely aligned with this first level of self-representation is body schema (Head and Holmes, 1911). In the present article, body schema is treated as the core of implicit bodily self-representation: a dynamic sensorimotor organization that supports posture, movement, coordination, the regulation of bodily boundaries, object reachability, spatial passability, and the adjustment of action to environmental properties and constraints (Bernstein, 1967; Gallagher, 1986; de Vignemont, 2010). In Gibsonian terms, this action-centered bodily organization is closely related to affordance perception: environmental possibilities for action are specified in relation to the organism’s bodily capacities, allowing rapid behavioral implementation when the relevant affordance is sufficiently clear (Gibson, 1979; Watson et al., 2002; Harley et al., 2009; Ravi et al., 2020; Wang et al., 2022).

For this reason, implicit bodily self-representation occupies an operationally borderline position. Ontologically, it belongs to the domain of self-representation because the organism regulates action with respect to its own bodily and agentive parameters (Gallagher, 2000; Wilson, 2002; de Vignemont, 2010). Operationally, however, it is difficult to distinguish reliably from body schema, online sensorimotor control, ecological calibration, and affordance-based regulation unless the animal’s own parameter can be shown to function as a discriminable task variable (Gibson, 1979; Warren and Whang, 1987; Heft, 1989). At this level, the body is represented primarily not as an object of awareness or as a perceptual “picture,” but as a system of action possibilities that enables the organism to move, orient, avoid obstacles, and relate its bodily parameters to the current situation.

A related but non-equivalent construct is body image, which is usually associated with more perceptual, phenomenological, and, in humans, conceptual, evaluative, social, and linguistically mediated forms of bodily representation; in the present framework, it is therefore treated as relevant to bodily self-representation, but not as equivalent to the implicit, action-guiding body schema (Schilder, 1935; Gallagher, 1986; de Vignemont, 2010).

Implicit bodily self-representation is primarily egocentric in organization: the organism’s own body functions as the reference point relative to which movement direction, object reachability, contact, resistance, spatial proximity, and manipulability are specified (Holmes and Spence, 2004; De Vignemont and Iannetti, 2015). This organization remains open to environmental and object-related transformations: local transformations may involve tools, objects, and extended action spaces while remaining anchored in ongoing bodily activity and organized around the body-in-action (Maravita and Iriki, 2004; Martel et al., 2016).

Thus, the first level provides the bodily sensorimotor basis of self-representation, but does not by itself provide sufficient operational grounds for inferring minimal self-related awareness. The next level emerges when the animal’s own body or action is not merely a background condition of online regulation, but a discriminable variable related to an environmental constraint in the anticipation of a possible behavioral outcome.

### 2.2 Minimal self-related awareness as self-referential anticipatory regulation

Minimal self-related awareness is understood here as a behaviorally detectable form of self–environment differentiation in which the individual takes its own body or action into account as a condition of possible behavioral outcomes. In a comparative context, this level does not require language, symbolic mediation, autobiographical narrative, introspective access, or a conceptual representation of oneself as an “I” (Gallagher, 2000), but becomes accessible through behavioral organization in which the organism relates itself to the environment, to other agents, or to constraints acting on its own body (Rochat, 2003; Lage et al., 2022).

At this level, affordances acquire self-referential and anticipatory significance. Affordance perception is already actor-relative: passability, reachability, supportability, or controllability are specified in relation to the bodily and agentive capacities of the organism (Gibson, 1979; Warren and Whang, 1987; Heft, 1989). Affordance-based regulation becomes relevant to self-representation when the behavioral contrast depends on the relation between a self-related parameter and an environmental constraint, rather than on the external stimulus configuration alone. Such parameters may include body size and shape (Brownell et al., 2007; Lenkei et al., 2020; Khvatov et al., 2023; Starić et al., 2026), weight (Brownell et al., 2007; Dale and Plotnik, 2017; Khvatov et al., 2021a), posture, flexibility, and mode of locomotion (Schiffner et al., 2014; Starić et al., 2026), object reachability (Ishak et al., 2008; Franchak and Adolph, 2014), manipulability (Dale and Plotnik, 2017; Vanhooland et al., 2025), and the degree of control over one’s own action and its sensory consequences (Synofzik et al., 2008; Kawabe et al., 2013; Haggard, 2017).

Minimal self-related awareness should be inferred from a convergent set of behavioral conditions that mark the transition from online sensorimotor regulation to self-referential anticipation of possible action:

1. Self-related parameter as a task variable. The relevant bodily or agentive parameter should be experimentally identifiable and variable across individuals, trials, or task configurations. This makes it possible to test whether behavior is organized by that parameter rather than by general body-scaled action alone (Machamer et al., 2000).
2. Relation between self-related parameter and environmental constraint. Behavioral choice should depend on the relation between the animal’s own parameter and an environmental constraint, such as body width and aperture size, body weight and support stability, limb length and object reachability, or action and sensory consequence (Warren and Whang, 1987; Brownell et al., 2007; Ishak et al., 2008; Synofzik et al., 2008; Dale and Plotnik, 2017). This relation, rather than an isolated external stimulus, makes the behavior self-representationally relevant.
3. Self-referential anticipation. The relevant anticipatory pattern appears when a bodily or agentive parameter is related to a possible outcome before final behavioral commitment (Pezzulo et al., 2015, 2016). The body functions here as a prospectively considered condition for selecting, delaying, modifying, or rejecting a possible action.
4. Epistemic adjustment before pragmatic commitment. Stronger interpretation is supported by epistemic actions aimed at obtaining or refining information about a self–environment relation, such as inspection, probing, delay, refusal, detour, repeated approach, or strategy change. Such actions can be distinguished from pragmatic actions that directly implement a selected outcome, in line with the distinction between uncertainty reduction and goal attainment (Kirsh and Maglio, 1994; Friston et al., 2015; Pezzulo et al., 2016; Parr and Friston, 2019).
5. Novelty, variability, and transfer. The task should include novel or variable configurations that weaken explanations based on habit, fixed response rules, or learning about specific stimuli (Harlow, 1949; Premack and Woodruff, 1978; Shettleworth, 2010). Transfer across positions, shapes, sizes, supports, or sensory conditions is especially informative when behavior remains tied to the same self-related parameter.
6. Controls against alternative explanations. Interpretation in terms of minimal self-related awareness requires controls for simpler explanations, including sensorimotor calibration, reinforcement history, ecological calibration, side bias, preference for larger openings, direct correction after failure, experimenter cueing, familiarity, habituation/dishabituation, and simple stimulus discrimination (Morgan, 1894; Shettleworth, 2010; Dickinson, 2012). In the absence of such controls, the behavior should be treated as suggestive rather than strong evidence.

This logic allows us to distinguish a weak affordance account from self-representational affordance evaluation. In a weak affordance account, the animal responds to a situation as passable, stable, reachable, or controllable without sufficient evidence that its own parameter functions as a task variable. In self-representational affordance evaluation, by contrast, behavior depends on the relation between a bodily or agentive parameter and the task constraint. A particularly informative pattern is a dissociation between broad or non-selective epistemic exploration and selective pragmatic commitment: for example, first approach may be distributed across several apertures, whereas first penetration attempts are directed toward the truly passable aperture (Lenkei et al., 2020; Khvatov, 2025).

This level should not be defined as a simple transition from egocentric to allocentric coding. In many cases, minimal self-related awareness may remain primarily egocentric: the organism only needs to evaluate whether it can pass through, reach, support, avoid, or move an object relative to its current bodily position (Holmes and Spence, 2004; De Vignemont and Iannetti, 2015). What is specific to this level is the capacity to relate an egocentric bodily model to object- or scene-based features of the environment, that is, to action possibilities specified not only by current sensorimotor calibration but also by the predicted outcome of a possible maneuver (Colby and Goldberg, 1999; Filimon, 2015; Wang et al., 2020).

### 2.3 Reflective self-representation and self-consciousness

At the third level, self-representation acquires a reflective form. In this sense, self-consciousness designates an organization of self-related processes in which the individual can represent itself not only as an acting body or behavioral agent, but also as an object of thought, evaluation, questioning, description, and interpretation (Gallagher, 2000; Rochat, 2003; Morin, 2006). The boundary between minimal self-related awareness and reflective self-representation lies where the self functions not only as a parameter of anticipated action, but also as an object of epistemic, symbolic, communicative, and socially mediated relation.

Reflective self-representation can be characterized by several interrelated features:

1. Self as an object of epistemic activity. At the level of minimal self-related awareness, epistemic actions primarily refine the relation between the body, action capacities, and the environment. At the reflective level, epistemic orientation can be directed toward the individual itself: the subject can inquire into, describe, evaluate, explain, and interpret itself (Gallagher, 2000; Morin, 2006).
2. Second-order relation to one’s own self-representation. The individual can relate not only to itself as a condition of action, but also to the way it is represented, understood, evaluated, or described. In this sense, self-representation becomes available for re-entry, revision, and incorporation into a more stable reflective organization of experience (Nelson, 2003).
3. Symbolic and communicative mediation. Reflective self-representation can be stabilized through signs, language, communication, and socially shared meanings. This feature is continuous with broader accounts of socially mediated higher psychological functions and shared intentionality (Leont’ev, 1978; Vygotsky,1978, 1997; Tomasello and Carpenter, 2007).
4. Narrative and temporal organization. The self can be organized across time through relations among past, present, and future, as well as through autobiographical and narrative forms of experience. Such organization allows the individual to return to itself as a relatively stable object of memory, anticipation, and interpretation (Habermas and Bluck, 2000; Nelson, 2003; Nelson and Fivush, 2004, 2020).
5. Normative and evaluative self-relation. At this level, self-related processes may include evaluation of oneself in relation to norms, roles, responsibility, success, failure, and the expectations of others. The self thereby participates in shared intentional, communicative, and normative structures within which self-evaluation, responsibility, social roles, and cultural identity become possible (Habermas and Bluck, 2000; Nelson and Fivush, 2004, 2020).
6. Distinction from enabling or interacting cognitive systems. Theory of mind, episodic memory, metacognition, empathy, narrative capacities, and social cognition may be deeply connected to self-related organization (Lage et al., 2022; Laurenzi et al., 2025). For comparative and operational purposes, their relevance to self-representation depends on whether they are independently specified as processes directed toward self-related aspects of the individual, including its body, actions, agency, temporal continuity, or social position (Gallagher, 2013; Birch et al., 2020).

Accordingly, bodily and minimal forms of self-representation in non-human animals should be treated as legitimate targets of comparative research, while remaining distinct from reflective self-consciousness in the stricter sense. The present article therefore focuses primarily on those forms of bodily and agentive self-representation that can be approached through behavioral organization, task structure, and control conditions, whereas reflective self-consciousness is discussed mainly as a conceptual boundary condition.

### 2.4 Interim conclusion: three levels of self-representation

Based on the preceding discussion, we use self-representation as an overarching term for different forms of organization of information about the organism, its body, actions, agency, and relation to the environment, through which the organism can take its own parameters into account in behavioral regulation. The levels distinguished below should not be understood as a simple evolutionary ladder, but as analytically distinct forms of self-representation that may be expressed mosaically across species, depending on their ecology, morphology, sensory systems, and behavioral demands (see Table 1).

1. Implicit bodily self-representation refers to an action-centered sensorimotor organization of the body, grounded in body schema and operating primarily in egocentric coordinates. At this level, the organism takes its own body into account directly in the course of action, but this form remains operationally difficult to distinguish from online sensorimotor control, ecological calibration, and affordance-based regulation unless the animal’s own parameter functions as a discriminable task variable.
2. Minimal self-related awareness refers to a behaviorally detectable form of self-referential anticipatory regulation in which the organism relates its own body, action capacities, or agency to environmental constraints as conditions of possible behavioral outcomes. At this level, epistemic and pragmatic actions may become temporally differentiated: the organism may first obtain or refine information about a self–environment relation and then use it to select, delay, modify, reject, or implement a self-scaled behavioral strategy.
3. Reflective self-representation refers to a higher-level form of self-representation in which the self becomes an object of epistemic, symbolic, communicative, social, and narrative organization. At this level, the individual can describe, evaluate, interpret, and narratively organize itself; in humans, this form of self-representation is strongly shaped by linguistic, cultural, normative, and socially shared structures.

**Table 1:** Three levels of self-representation in comparative research.

Analytical parameter	Implicit bodily self-representation	Minimal self-related awareness	Reflective self-representation/self-consciousness
Core form of self-representation	Action-centered sensorimotor organization of the body, grounded in body schema (Head and Holmes, 1911; Gallagher, 1986; de Vignemont, 2010) and supporting posture, movement, bodily boundaries, and body–environment coupling.	Self-referential anticipatory regulation in which the animal’s own body, action, or agency functions as a condition of possible behavioral outcomes (Rochat, 2003; Brownell et al., 2007; Lage et al., 2022).	Reflective organization in which the self can become an object of thought, evaluation, questioning, description, interpretation, and narrative organization (Gallagher, 2000; Morin, 2006; Nelson and Fivush, 2004).
Operational status	Operationally borderline: the organism regulates action with respect to its own bodily and agentive parameters, but this level is difficult to distinguish from body schema, online sensorimotor control, ecological calibration, and affordance-based regulation (Gibson, 1979; Warren and Whang, 1987; Heft, 1989).	Operationally accessible through behavioral contrasts showing that a self-related parameter is related to an environmental constraint under appropriate controls (Brownell et al., 2007; Dale and Plotnik, 2017; Lenkei et al., 2020).	Operationally accessible primarily through reflective, symbolic, communicative, narrative, and socially mediated forms of self-relation (Leont’ev, 1978; Vygotsky, 1978; Tomasello and Carpenter, 2007); in non-human animals, only precursors or partial analogs can usually be assessed.
Degree of differentiation/integration	Low differentiation and high integration: bodily and environmental parameters are coupled within ongoing sensorimotor regulation.	Partial differentiation: bodily or agentive parameters become functionally separable as task variables and can be related to constraints before behavioral commitment.	High differentiation followed by symbolic, social, and narrative reintegration: the self can be described, evaluated, interpreted, and organized within relatively stable systems of meaning.
Anticipatory organization	Anticipation is embedded in online action regulation and expressed as immediate sensorimotor adjustment to the current situation.	Anticipation is self-referential: the animal relates its own bodily or agentive parameter to a possible outcome before selecting, delaying, modifying, or rejecting an action.	Anticipation extends to temporally distant, socially mediated, and symbolically organized consequences, including future selves, norms, roles, responsibility, and autobiographical continuity.
Epistemic and pragmatic actions	Epistemic and pragmatic aspects are largely fused: information about the body–environment relation is updated through ongoing action itself.	Epistemic and pragmatic actions may become temporally differentiated (Kirsh and Maglio, 1994; Friston et al., 2015; Pezzulo et al., 2016): inspection, probing, hesitation, refusal, or sensory sampling may precede passage, detour, support choice, action correction, or other forms of behavioral commitment.	Epistemic activity can be directed toward the self as such; pragmatic action may be regulated by self-description, self-evaluation, social norms, and narrative organization of experience.
Typical behavioral expressions	Postural adjustment, movement coordination, obstacle avoidance, reachability calibration, body-scaled affordance use (Gibson, 1979; Warren, 1984; Warren and Whang, 1987), and online correction of action.	Passability judgments, body-as-obstacle tasks, weight/support tasks (Brownell et al., 2007; Dale and Plotnik, 2017; Khvatov, 2025), agency-control paradigms, self-scaled action selection, and dissociations between broad epistemic exploration and selective pragmatic commitment.	Self-description, self-evaluation, autobiographical narration (Habermas and Bluck, 2000; Nelson and Fivush, 2004, 2020), role-taking, responsibility, reflective self-interpretation, and self-organization within shared meanings and norms.
Main alternative explanations/limits	Body schema, sensorimotor control, ecological calibration, affordance perception, and online movement correction may explain behavior without requiring a stronger inference to self-representation.	Simpler explanations include sensorimotor calibration, reinforcement history, ecological calibration, side bias, preference for larger openings, direct correction after failure, experimenter cueing, familiarity, habituation/dishabituation, and simple stimulus discrimination (Morgan, 1894; Shettleworth, 2010; Dickinson, 2012).	Theory of mind, episodic memory, metacognition, empathy, narrative capacities, and social cognition may interact with self-related organization without automatically constituting reflective self-representation.
Comparative-psychological status	The most basic and widely distributed bodily-sensorimotor basis of self-representation; important but weakly discriminable as evidence for self-representation in a stronger operational sense.	The central level for comparative research on animal self-representation, because it can be operationalized through behavioral tasks without requiring language, reflective self-report, or symbolic self-description (de Waal, 2019; Lage et al., 2022; Laurenzi et al., 2025).	Most fully developed in humans in the strict sense; in non-human animals, comparative research may address precursors, partial analogs, or interacting mechanisms rather than fully reflective self-consciousness.

The table summarizes three analytically distinguished levels of self-representation: implicit bodily self-representation, minimal self-related awareness, and reflective self-representation or self-consciousness. The first level refers to action-centered bodily organization grounded in body schema and online sensorimotor regulation; it is ontologically related to self-representation but remains operationally difficult to distinguish from body-scaled affordance regulation unless the animal’s own parameter functions as a discriminable task variable. The second level refers to self-referential anticipatory regulation, in which bodily or agentive parameters are related to environmental constraints before final behavioral commitment. The third level refers to reflective forms in which the self becomes an object of epistemic, symbolic, communicative, social, and narrative organization. The table is intended as a conceptual guide for comparative research rather than as a strict evolutionary ladder.

This three-level distinction does not yet specify how each level should be experimentally investigated. Rather, it provides the conceptual vocabulary required to evaluate existing paradigms and to develop, in the following sections, a modal-modular and operational framework of animal self-representation.

## 3 The modal-modular model of animal self-representation

### 3.1 Self-representation as a functional regulatory architecture

The previous section distinguished three levels of self-representation: implicit bodily self-representation, minimal self-related awareness as anticipatory bodily and agentive self-representation, and reflective self-representation, or self-consciousness. The present section translates this vertical distinction into the functional architecture of the MMM. The model analyzes self-representation in terms of sensory modalities, bodily and agentive parameters, and motivational-behavioral orientations, that is, the channels, parameters, and behavioral contexts through which the body, action, and agency become relevant to behavioral regulation. In this sense, the MMM shifts the analysis from a unitary question of self-awareness to the specification of sensory access, task context, target parameters, and evidential strength (Rochat, 2003; Bekoff and Sherman, 2004; Birch et al., 2020).

In ecologically organized behavior, animals relate objects and events in the environment to their own bodily capacities, limitations, and action parameters. This logic is compatible with an affordance-based view, according to which possibilities for action are specified in relation to the body and motor capacities of the actor (Gibson, 1979; Warren, 1984; Warren and Whang, 1987). The MMM narrows the self-representational interpretation to cases in which the decisive behavioral contrast depends on the relation between a self-related parameter and a task constraint. Such an interpretation is warranted when behavior is organized not only by the external stimulus configuration, a simple preference, reinforcement history, or a previously learned motor routine, but by parameters of the animal itself, such as body size, body weight, bodily boundaries, own signals, or controllability. Accordingly, self-representation as a regulatory architecture should be described through the functional dimensions in which body, action, and agency become operationally distinguishable behavioral variables.

### 3.2 Levels, modules, and functional dimensions

The relation between levels and modules in the MMM should be understood as a relation between the degree of organization of self-representation and the differentiation of its functional components. Levels describe the vertical organization of self-representation, from implicit bodily regulation to minimal anticipatory self-related awareness and reflective self-consciousness. Modules specify the horizontal functional architecture through which particular self-related parameters become behaviorally expressed in recurrent classes of tasks. In this sense, functional dimensions and candidate modules operationalize the three-level distinction by showing through which sensory channels, bodily or agentive parameters, and motivational-behavioral contexts self-representation becomes accessible to comparative research.

**Figure 1:**
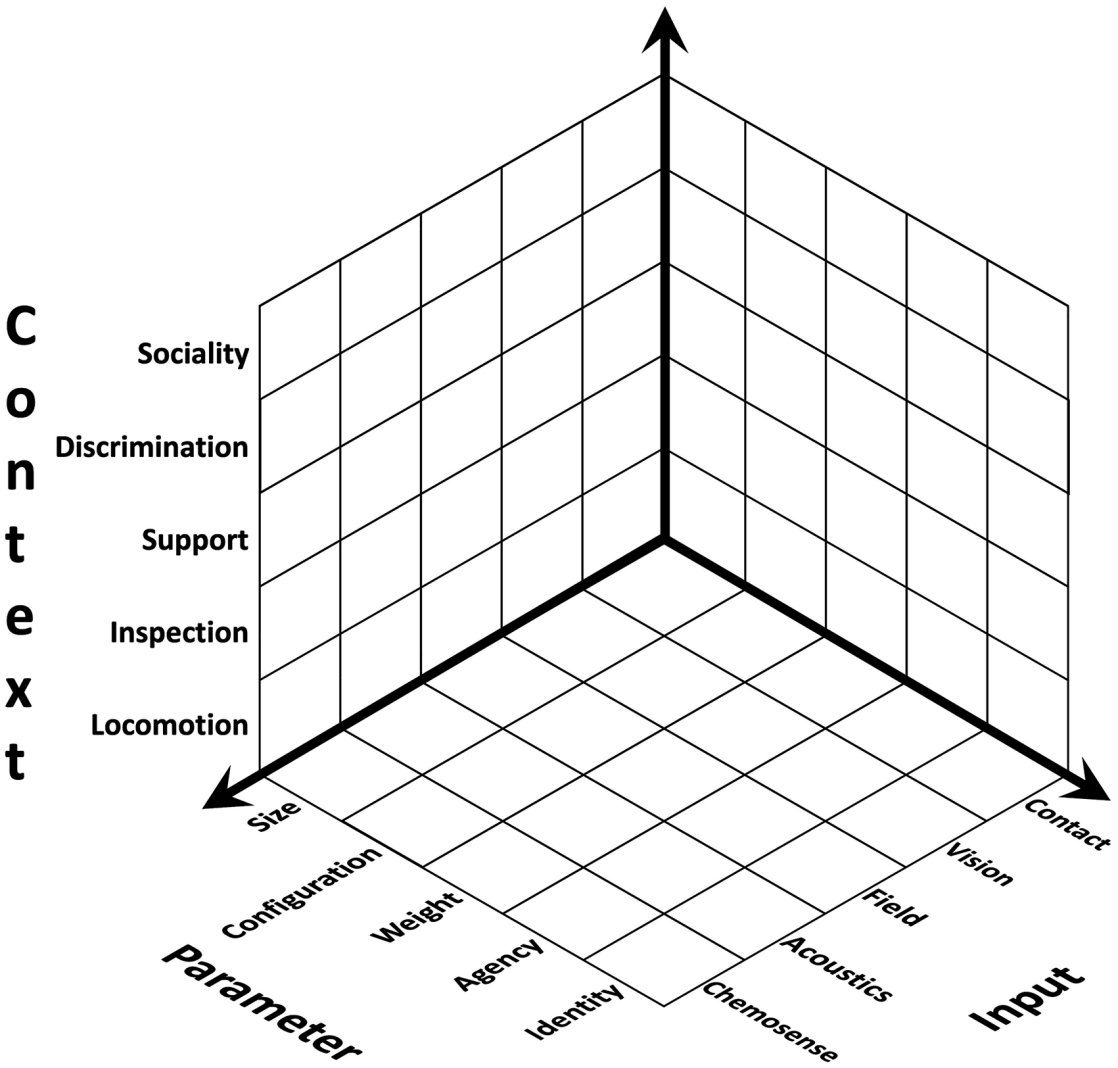
Categorical coordinate space of the Modal-Modular Model of Animal Self-Representation. The model represents animal self-representation as a three-dimensional categorical space defined by three functional dimensions: Input, Parameter, and Context. Here, Input is a condensed label for sensory input, Parameter for represented bodily or agentive parameter, and Context for motivational-behavioral orientation. Each axis denotes not a quantitative scale, but a set of analytically distinguishable categories used to describe how self-related information becomes available to the organism, which bodily or agentive properties are represented, and in which motivational-behavioral domain these properties become functionally relevant. The ordering of categories along each axis is schematic and arranged to group functionally adjacent module components; it does not imply metric distances, developmental stages, evolutionary sequence, or hierarchical relations. Input refers to major classes of sensory access through which information about the organism’s own body, actions, or action consequences becomes available. Contact includes contact-based and near-body forms of sensory access, such as tactile, proprioceptive, vestibular, vibrissal, and other sensorimotor inputs directly related to body position, movement, and boundaries. Vision refers to visual access to the body, reflection, appearance, spatial layout, and visually specified body-environment relations. Field refers to field-mediated or medium-dependent forms of sensory access, such as hydrodynamic cues mediated by the lateral line, electric sensing where applicable, pressure-based cues, or other species-specific physical signals through which an animal may obtain information about its own movement, bodily position, or action consequences. Acoustics includes auditory and vibratory channels, including self-generated vocalizations, acoustic consequences of action, and substrate-borne vibratory signals. Chemosense refers to olfactory and other chemical cues related to the organism’s own body, individual odor profile, or chemically mediated forms of self-other discrimination. Parameter refers to the represented bodily or agentive property that becomes relevant for behavioral regulation. Size denotes relatively stable dimensional properties of the body, including body size and shape as constraints on possible action. Configuration refers to the current bodily arrangement, including posture, flexibility, body boundaries, and dynamically changing bodily form in action. Weight denotes body weight, load-bearing relations, and situations in which the animal’s own mass becomes a condition for successful or unsuccessful task performance. Agency refers to action control, authorship of action, and the discrimination of self-generated consequences from externally caused changes. Identity refers here not to reflective personal identity, but to individual-specific self-signal or appearance-related parameters through which the individual’s body or signals can be distinguished from those of others, including appearance, odor, voice, electric, acoustic, chemical, or other species-specific signal profiles. Context refers to the motivational-behavioral domain in which self-related information becomes functionally relevant and empirically accessible through behavior. Locomotion includes movement through space, obstacle negotiation, and passability judgments in relation to the animal’s own body. Inspection refers to self-directed exploration or examination of the body, appearance, reflection, or modified self-related signal. Support includes support use, load-bearing situations, manipulation, and object-related interactions in which the body, its weight, or its position constrains task success. Discrimination refers to tasks involving the differentiation of own versus other signals, self-generated versus externally generated effects, or the animal’s own body versus other objects or agents. Sociality denotes social, communicative, and interactive contexts in which self-representation may contribute to self-other differentiation, individual recognition, action coordination, or socially embedded forms of behavior.

The MMM describes self-representation along three interconnected functional dimensions, schematically represented in Figure 1. The term “modal” is used here in two senses: in the narrower sense of sensory modality, and in a broader functional sense referring to the mode through which self-related information becomes available, organized, and behaviorally expressed. These dimensions distinguish sensory access, represented parameter, and behavioral context, since the same bodily or agentive parameter may be represented through different sensory systems, whereas the same sensory channel may contribute to different forms of self-related regulation.

1. Input refers to the sensory access through which information about the animal’s own body, actions, and their consequences becomes available for behavioral regulation. Depending on morphology, phylogenetic history, lifestyle, and ecological niche, relevant input may include visual, tactile, proprioceptive, vestibular, auditory, olfactory, vibrissal, hydrodynamic, electric, pressure-based, or other species-specific cues (Dangles et al., 2009; Stevens, 2013; Oteiza and Baldwin, 2021). For this reason, sensory access should be specified for each species and task rather than derived from human-centered or visually oriented models (de Waal, 2019; Lage et al., 2022).
2. Parameter refers to the bodily or agentive property that becomes functionally relevant in a given task. Such parameters may include body size, body shape, body boundaries, body weight, posture, flexibility, reachability, controllability, authorship of action, visible appearance, own odor, own voice, or the sensory consequences of self-generated movements (Brownell et al., 2007; Synofzik et al., 2008; de Vignemont, 2010).
3. Context refers to the motivational-behavioral domain in which a particular parameter matters for action (Gibson, 1979; Kirsh and Maglio, 1994). In many forms of animal minimal self-related awareness, self-representation remains embedded in object-directed behavior: the animal takes its own body into account in order to pass, reach, avoid, support, manipulate, or control (Rochat, 2003; Lage et al., 2022). In reflective forms of self-representation, characteristic primarily of the third level, one’s own body, action, or appearance may become the object of explicit attention, exploration, evaluation, modification, or narrative integration (Gallagher, 2000).

In actual behavior, these dimensions are usually integrated within recurrent task classes. Such relatively stable integrations constitute functional modules of self-representation, to which we now turn.

### 3.3 Candidate modules and their evidential status

If the three functional dimensions of the MMM analytically decompose self-representation into sensory input, represented bodily or agentive parameter, and motivational-behavioral context, the notion of a module refers to their relatively stable functional integration. In the present framework, a module of self-representation is understood as a relatively autonomous functional subsystem through which an organism uses information about its own body, actions, or agentive capacities to solve a recurrent class of behavioral problems. In this sense, modules do not designate isolated “parts” of self-representation, but stable modes of behavioral organization in which a particular self-related parameter becomes relevant to action (Thelen and Smith, 1994; van Opstal, 1996).

The notion of a module is used here in a functional rather than a strictly Fodorian sense: modules of self-representation may be relatively autonomous, domain-specific, and behaviorally identifiable without being rigidly encapsulated, anatomically localized, innately fixed, or universal across species (Fodor, 1983). In this respect, they are closely related to the Gibsonian logic of affordances, because they concern possibilities for action specified in relation to the bodily and agentive parameters of a particular organism (Gibson, 1979; Warren and Whang, 1987). Within the MMM, a module becomes self-representationally relevant when behavior is organized around the relation between a self-related parameter and a task constraint: an aperture is passable or impassable, a support stable or unstable, and an event controllable or uncontrollable relative to the body and action capacities of a given animal. Such a module is closer to a functional organ than to a morphologically discrete structure: it may include sensory, motor, motivational, and regulatory components that are temporarily or stably assembled around a particular task within the individual–environment system (Nardi, 1996; Sokolova, 2011).

It follows that the MMM treats modular profiles as species-specific rather than as a universal set of components expressed in the same way across all animals (Bekoff and Sherman, 2004; de Waal, 2019). The same candidate module may vary in degree of expression, rely on different sensory systems, and combine with other modules in different ways. Mixed profiles are therefore expected, because analytically distinguishable modules may be behaviorally coupled in task-specific ways. The present version outlines five candidate domains: body size/passability, body weight/support, agency/action-control, appearance-related self-representation, and modality-specific self-signal recognition. These domains are provisional and operationally defined; they serve as examples of a modular architecture rather than as an exhaustive taxonomy of animal self-representation. Their evidential status is also unequal: body size/passability and body weight/support currently provide the clearest operational cases, whereas agency/action-control, appearance-related, and self-signal domains require more cautious interpretation and specific controls. Detailed criteria for strong, suggestive, and insufficient evidence, as well as control conditions and validation routes, are presented in Section 4 and Supplementary Table 1.

#### 3.3.1 Body size/passability module

**Figure 2:**
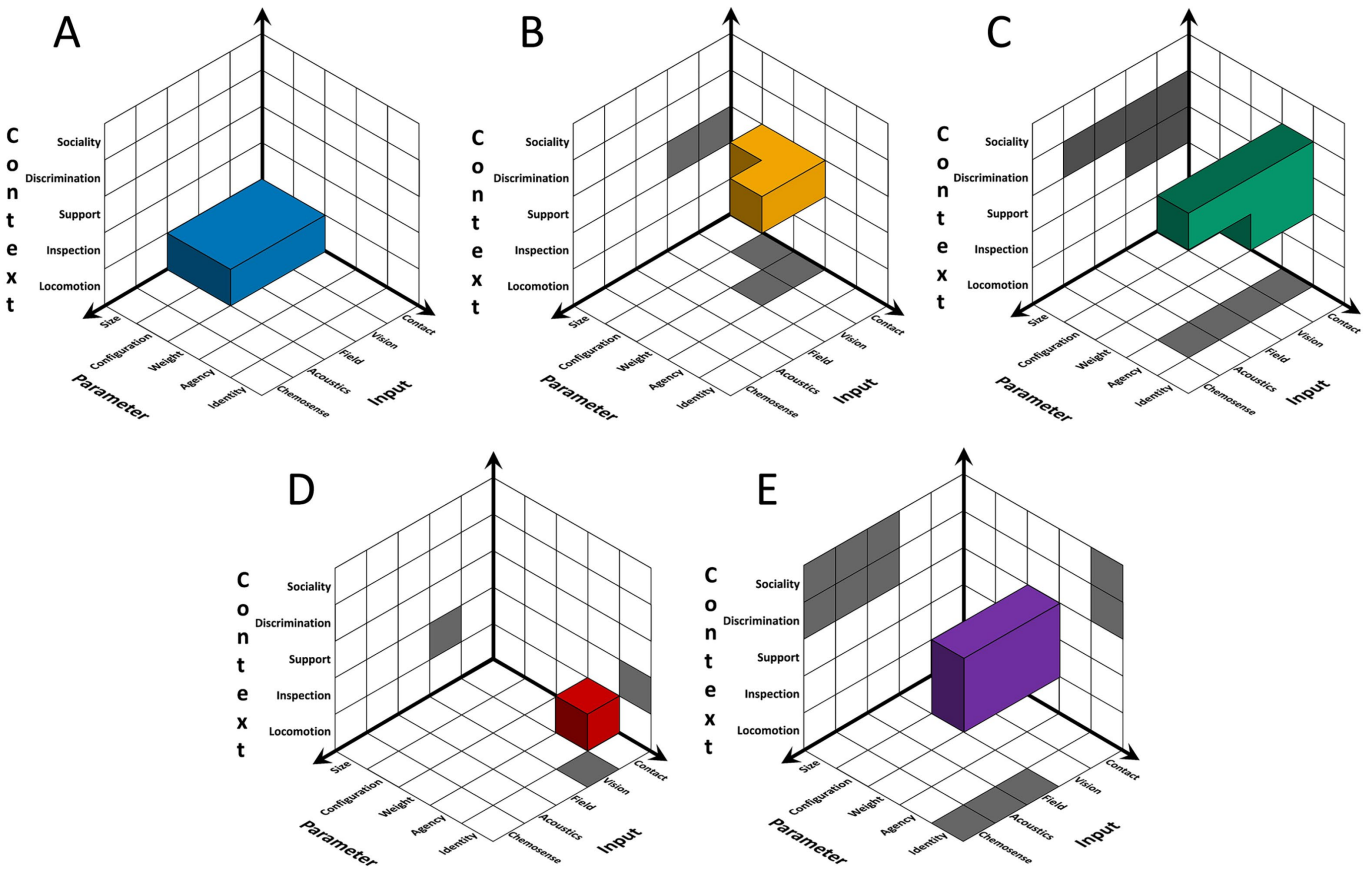
Candidate modules of animal self-representation within the categorical coordinate space of the Modal-Modular Model. Each panel represents one candidate module, or a group of closely related candidate modules, as a colored region within the space defined by Input, Parameter, and Context. Filled blocks do not indicate quantitative coordinates, but schematic combinations of sensory access, represented bodily or agentive parameter, and motivational-behavioral orientation through which the corresponding module can be operationally defined and experimentally tested. Gray projection areas are included only as visual guides for locating each module relative to the three coordinate planes. **(A)** Blue region: body size/passability module. This module occupies the region in which contact-based, visual, and field-mediated forms of sensory access are linked to body size and bodily configuration in the context of locomotion and passability. It corresponds to tasks in which an animal must take its own dimensions, shape, boundaries, or current bodily arrangement into account when passing through apertures, selecting routes, avoiding obstacles, seeking shelter, pursuing prey, or moving through constrained substrates. In this sense, the module represents bodily self-representation as self-scaled action, that is, behavioral regulation based on the relation between the animal’s own bodily parameters and the spatial constraints of the environment. **(B)** Yellow-orange region: body weight/support module. This module is centered on combinations linking contact-based sensory access, weight- and support-related parameters, and contexts of support, load-bearing, and manipulation. It describes situations in which the animal’s own mass, bodily pressure, balance, relation to a supporting surface, or bodily position relative to an object becomes a condition for successful action. This module includes body-as-obstacle, support-use, and load-bearing paradigms in which the animal must take into account that its own body is not only an acting body in the environment, but may also function as a source of obstruction, load, or physical constraint. **(C)** Green region: agency/action-control module. This module occupies the region associated with agency, action control, and the discrimination of self-generated consequences from externally caused changes. It primarily includes contact-based, visual, and field-mediated sensory access, including proprioceptive, tactile, and other action-contingent feedback. The field-mediated portion of the region should be understood as a possible species-specific extension for cases in which medium-dependent physical cues carry information about self-generated action consequences. Unlike more localized bodily modules, this module has a distributed character, since agency may be expressed through action support, effect control, movement monitoring, self-generated versus externally generated outcome discrimination, and more complex forms of contingency between action and consequence. **(D)** Red region: appearance-related module. This module is represented as a compact region linking visual input, an identity-related parameter, and the context of inspection. Its compact size is intentional and reflects the restricted operational scope of visual appearance-related self-representation within the MMM: mirror self-recognition is treated here as one specific, modality-constrained component rather than as a privileged endpoint or universal criterion of animal self-representation. It corresponds to appearance-based forms of self-representation, including mirror self-recognition and other tasks in which visible appearance, reflection, or a visually accessible modification of the body becomes the object of directed exploration. **(E)** Purple region: modality-specific self-signal recognition modules. This region is broader than the Appearance-related module and corresponds not to a single already established mechanism, but to a provisional group of potential signal-specific subsystems concerned with distinguishing one’s own signals, traces, or sensory consequences from those of other individuals or environmental events. It includes possible olfactory, acoustic, vibratory, chemical, field-mediated, tactile, hydrodynamic, or multimodal forms of own-signal recognition, depending on the sensory ecology of the species. Accordingly, this region should be read not as a finalized taxonomic unit, but as a working domain for future differentiation, in which distinct sensory channels may support different forms of own-signal recognition, self-other discrimination, agency-related monitoring, or individual-specific regulation.

The body size/passability module concerns behavioral regulation in tasks in which an animal relates its body dimensions, shape, boundaries, or flexibility to a spatial constraint, such as an aperture, passage, obstacle, or other environmental configuration (Figure 2A). Its guiding question is: can this body pass through this space? This domain has a clear operational structure because the self-related parameter can be directly related to a measurable task constraint (Brownell et al., 2007; Khvatov et al., 2023; Pongrácz, 2024; Khvatov, 2025; Vanhooland et al., 2025).

- Sensory input: visual, tactile, proprioceptive, vestibular, vibrissal, hydrodynamic, or other near-field mechanosensory cues that allow the body to be related to the geometry of an obstacle.
- Represented parameters: body width, height, length, diameter, boundaries, shape, flexibility, and possible bodily deformation during passage through an aperture or spatial gap.
- Motivational-behavioral orientation: passage, shelter seeking, obstacle negotiation, danger avoidance, prey pursuit, route selection, or movement through complex substrates; the animal organizes action relative to its own bodily parameters.

#### 3.3.2 Body weight/support module

The body weight / support module concerns behavioral regulation in tasks in which an animal relates body mass, load, balance, or pressure to support relations, object movement, or physical constraints on action (Figure 2B). Its guiding question is: can this body be supported, balanced, moved, or removed as an obstacle under these conditions? This domain is close to body size/passability, but has a distinct operational logic because the task constraint is defined by support, load, pressure, and bodily obstruction rather than by spatial passability (Brownell et al., 2007; Moore et al., 2007; Dale and Plotnik, 2017; Khvatov et al., 2021a; Vanhooland et al., 2025).

- Sensory input: proprioceptive, vestibular, tactile, and pressure-related signals, visual assessment of support, motor feedback during pushing, pulling, climbing, or balancing, and auditory or vibrational cues indicating support instability.
- Represented parameters: body mass, pressure, balance, load, support relations, the body as an obstacle to action, and the relation between body weight and the possibility of moving an object.
- Motivational-behavioral orientation: standing, climbing, balancing, moving objects, reaching a goal when one’s own body blocks the action, avoiding falls or support collapse, and manipulating objects under bodily constraint.

#### 3.3.3 Agency/action-control module

The agency/action-control module concerns behavioral regulation in tasks in which an animal relates its own action to its sensory or environmental consequences (Figure 2C). Its guiding question is: is this event produced, controlled, or modifiable by my own action? This domain is theoretically central to self-representation, but requires especially cautious interpretation because behavior in such tasks may overlap with associative learning, motor control, and general sensitivity to causal relations (Synofzik et al., 2008; Kaneko and Tomonaga, 2011; Haggard, 2017; Terao et al., 2026).

- Sensory input: proprioceptive, tactile, visual, and auditory feedback, efference-related signals, temporal contingency between action and effect, and mismatch between expected and actual sensory consequences of action.
- Represented parameters: authorship of action, controllability, action-effect contingency, self-generated movement, expected sensory consequences, and the distinction between self-generated action and external causation.
- Motivational-behavioral orientation: manipulation, object or tool use, control over environmental changes, action correction, movement monitoring, exploration of controllable versus uncontrollable events, and, in more complex cases, socially directed forms of action.

#### 3.3.4 Appearance-related module

The appearance-related module concerns behavioral regulation in tasks in which an animal relates the visible appearance of the body to its own body, especially through a mirror, image, or another form of visual mediation (Figure 2D). Its guiding question is: does this visible appearance belong to my own body, and does it contain a behaviorally relevant change? This domain is well developed in the mirror self-recognition tradition, but in the MMM it is treated as a specific visually mediated component rather than as a universal index of self-representation as a whole (Bekoff and Sherman, 2004; de Waal, 2019; Lage et al., 2022; Priestley and Wascher, 2026).

- Sensory input: primarily visual information, and, in more developed forms, its integration with tactile and proprioceptive body mapping, sensorimotor contingency, memory of one’s usual appearance, and socially mediated visual feedback.
- Represented parameters: the visible body surface, body parts accessible through reflection or external imaging, marks or changes in appearance, body configuration from an external perspective, and, in humans, appearance as a socially and symbolically evaluated parameter.
- Motivational-behavioral orientation: inspection, grooming, mark-directed behavior, removal of foreign material, sensorimotor contingency checking, and, in more developed forms, self-evaluation, social self-presentation, symbolic identity, body image, self-concept, and self-for-others.

#### 3.3.5 Modality-specific self-signal recognition modules

Modality-specific self-signal recognition modules refer to a group of potential signal-specific subsystems concerned with distinguishing one’s own odors, voices, traces, or other sensory consequences from signals produced by other individuals or by the environment (Figure 2E). Their guiding question is: does this odor, sound, trace, or signal correspond to my own body, my own signal profile, or my individual identity? In the MMM, these subsystems require cautious interpretation: own-signal discrimination becomes relevant to self-representation when the animal uses the signal to regulate behavior in relation to its own body, action, agency, or individual identity, rather than merely responding to familiarity, novelty, or general signal discriminability (Kaplan et al., 2008; Horowitz, 2017; Burghardt et al., 2021; Cazzolla Gatti et al., 2021; Szabo and Ringler, 2023).

- Sensory input: olfactory, auditory, vibrational, tactile, chemical, hydrodynamic, or multimodal signals, depending on which self-produced traces and communication channels are ecologically relevant for a given species.
- Represented parameters: own odor, own voice, individual chemical signature, self-produced acoustic signal, bodily trace, action-generated signal, or modified own signal.
- Motivational-behavioral orientation: territorial behavior, social and individual recognition, kin or group discrimination, mate choice, detection of contamination or foreign material, vocal monitoring, communicative control, and distinguishing self-generated from externally generated sensory consequences.

The set of candidate modules outlined above should be distinguished from broader systems of self-related cognition (Gallagher, 2013; Birch et al., 2020; Laurenzi et al., 2025). Episodic memory, theory of mind, metacognition, empathy, social cognition, or narrative organization may rely on self-representation, transform it, or integrate it into more complex architectures, but they qualify as modules of self-representation only when they directly represent parameters of one’s own body, action, agency, or individual identity. This restriction delimits the comparative and operational scope of the MMM: the model treats self-representation primarily as the capacity to use information about one’s own body, actions, and agentive capacities to regulate behavior under ecologically relevant constraints. The next step is to translate this architecture into operational criteria, behavioral markers, control conditions, and alternative explanations that should be considered when experimentally investigating self-representation in animals.

## 4 Operationalizing animal self-representation: experimental approaches and behavioral criteria

### 4.1 General principles of operationalization

This section translates the modal-modular architecture into operational criteria for comparative research. Experimental paradigms are treated as module-specific procedures targeting particular self-related parameters under defined sensory, motivational, and behavioral conditions, thereby providing complementary routes for comparative analysis (de Waal, 2019; Lage et al., 2022; Vanhooland et al., 2025).

#### 4.1.1 Principles for operationalizing and validating self-representation modules

Several interconnected methodological principles are required for operationalizing and validating candidate modules of self-representation in animals. These principles provide a general route from the modal-modular architecture to experimental tasks, quantifiable behavioral contrasts, evidence levels, and controls for alternative explanations.

1. Principle of modality matching. The experimental task should correspond to the sensory channels through which a given species normally obtains behaviorally relevant information about its own body, actions, or individual signals (Dusenbery, 1992; Shettleworth, 2001; Stevens, 2013; Bräuer et al., 2020). Visual tests may be informative for visually oriented species, whereas animals whose behavior primarily relies on olfaction, audition, tactile sensitivity, vibrissal sensing, hydrodynamic perception, or electroreception require procedures matched to the corresponding modality-specific routes of access.
2. Principle of behavioral and ecological relevance. A paradigm becomes informative when the tested self-related parameter is functionally relevant within the ordinary behavioral ecology of the species (Tinbergen, 1963; Shettleworth, 2001; Bräuer et al., 2020; Horn et al., 2022). Self-representation becomes behaviorally expressed in situations in which bodily, agentive, or individual-signal parameters operate as conditions for successful action, such as moving through constrained spaces, selecting adequate support, manipulating objects, distinguishing self-produced from externally produced action consequences, or responding to individually meaningful signals.
3. Principle of functional module separation. Evidence obtained in a given paradigm should be assigned to the functional module that the paradigm actually targets (Povinelli and Cant, 1995; Bekoff and Sherman, 2004; Lenkei et al., 2020; Lage et al., 2022; Laurenzi et al., 2025). In the MMM, modularity is understood in a functional rather than strictly anatomical, encapsulated, or domain-hardwired sense. A candidate module should be specified by a combination of features: a target self-related parameter, a behavioral function, relevant sensory channels, within-module convergence across tasks, transfer within the same parameter domain, and possible dissociations from other components. Thus, a positive result in MSR primarily supports appearance-related self-representation, whereas a negative result in the mirror test does not rule out body-size/passability-related, body-weight/support-related, agency-related, or modality-specific self-signal forms of self-representation.
4. Principle of epistemic–pragmatic differentiation. Behavioral analysis should distinguish epistemic actions, which serve to obtain or refine information about the relation between the animal’s body, action capacities, and task constraints, from pragmatic actions, which directly implement a selected behavioral outcome (Kirsh and Maglio, 1994; Friston et al., 2015; Pezzulo et al., 2016). Final success or failure should therefore be interpreted together with the structure of the behavioral sequence, including what the animal first investigates, which task-relevant features it samples, and how it subsequently commits to an attempt, refusal, correction, or choice.
5. Principle of quantifiable behavioral contrasts. The MMM operationalizes self-representation by specifying module-specific behavioral contrasts that can be quantified within each candidate module. This contrast-based strategy provides a more appropriate basis for cross-species comparison than a single global numerical index, given the diversity of sensory systems, body plans, ecological demands, and task affordances across taxa. Such contrasts may include passable versus non-passable apertures, first approach versus first penetration attempt, adequate versus inadequate support, body-on-object versus body-off-object conditions, controllable versus uncontrollable outcomes, own versus modified-own signals, and immediate versus delayed or distorted feedback. They can be measured through latencies, frequency and duration of inspection, first choices, first attempts, refusals, corrections, first-attempt success, transfer to novel configurations, and changes in behavior under control conditions (Brownell et al., 2007; Dale and Plotnik, 2017; Lenkei et al., 2020; Khvatov et al., 2023, 2024).
6. Principle of first-trial performance, transfer, and control. The strength of the evidence depends on whether the animal shows self-scaled behavior only after learning or already on the first or early trials, and whether the solution transfers to novel stimuli, configurations, positions, shapes, supports, or feedback conditions (Heyes, 1994; Shettleworth, 2010; Dickinson, 2012; Zentall et al., 2014). The strongest evidence is obtained when behavior remains structured by the relation between the animal’s own bodily or agentive parameter and the task constraint while remaining robust under controls for position, size, odor, novelty, trial order, social responses, tactile irritation, motivation, and other task-irrelevant features.
7. Principle of evidence grading and conservative interpretation. Behavioral patterns should be interpreted not only in terms of whether a relevant effect is present, but also in terms of their evidential status as suggestive, strong, or insufficient evidence (Gallagher, 2013; Birch et al., 2020; Crump and Birch, 2022; Priestley and Wascher, 2026). Adaptive behavior alone should not be automatically interpreted as self-representation. A self-representational interpretation becomes stronger when the behavioral pattern is linked to the target self-related parameter, expressed in quantifiable contrasts, robust under controls for alternative explanations, and, where predicted, transferable to novel configurations. When associative learning, sensorimotor calibration, affordance perception, ecological calibration, novelty, habituation/dishabituation, familiarity, reward preference, or simple stimulus discrimination fully explain the result, the evidence should be treated as suggestive or insufficient rather than strong.

#### 4.1.2 A standard evidence-graded template for module-specific analysis

Based on these principles, and following the specification of target parameters, sensory access, and species-specific relevance in Section 3.5, the following analysis focuses on the operationalization and validation of the candidate modules (Birch et al., 2020; Crump and Birch, 2022; Laurenzi et al., 2025). Each candidate module is therefore examined according to a common evidence-graded template.

1. Main task families: by which paradigms can the module be tested? For each module, the main classes of experimental tasks should be identified through which the corresponding self-related parameter can be made behaviorally observable.
2. Quantifiable behavioral contrasts and dependent variables: what is compared and measured? The analysis should specify the relevant contrasts between experimental conditions and the corresponding behavioral measures, including latencies, approaches, inspection, contacts, attempts, choices, refusals, corrections, avoidance, first-attempt success, transfer, or self-directed behavior.
3. Epistemic and pragmatic actions: which actions acquire information, and which actions implement the selected outcome? Particular attention should be given to the behavioral sequence, insofar as it allows actions aimed at refining the relation between the body, action, and task constraint to be distinguished from actions directly implementing a behavioral choice.
4. Evidence levels: what counts as suggestive, strong, or insufficient evidence? For each module, the criteria that justify a given evidential status should be stated explicitly, indicating how securely a behavioral pattern supports the corresponding self-representational interpretation.
5. Alternative explanations and control conditions: which simpler mechanisms must be excluded? For each module, alternative explanations that may fully account for the result without invoking self-representation should be specified, including learning, novelty, sensory salience, positional preferences, irritation, social responses, chance success, training effects, odor trails, habituation/dishabituation, reward preference, and other uncontrolled features of the experimental situation (Heyes, 1994; Shettleworth, 2010; Dickinson, 2012; Zentall et al., 2014).
6. Validation routes, dissociations, and profile contribution: how can the module be validated, separated from other components, and incorporated into a species-specific modal-modular profile? Validation should consider within-module convergence across tasks, transfer within the same parameter domain, robustness under control conditions, and possible dissociations between modules.

This template does not yet constitute a computational model, but it provides a pre-computational operational architecture for future formalization. At a general level, each profile can be described as a relation among a self-related parameter, an environmental constraint, a sensory modality, a task demand, and a behavioral outcome.

This logic is summarized in condensed form in Table 2 and implemented in full in the revised Supplementary Table 1. The latter also makes explicit that candidate modules may differ in their current evidential status, with some domains being relatively well supported and others requiring further validation. Together, Table 2 and Supplementary Table 1 provide an evidence-graded, profile-based operational scaffold for the candidate modules of the MMM, including task families, quantifiable behavioral contrasts, dependent variables, epistemic and pragmatic actions, evidence levels, alternative explanations, control conditions, validation routes, supporting and weakening patterns, expected dissociations, and contributions to species-specific modal-modular profiles (Crump and Birch, 2022; Priestley and Wascher, 2026).

**Table 2:** Condensed operational matrix for candidate modules of animal self-representation.

Candidate module	Target parameter and task families	Quantifiable contrasts/variables	Evidence and controls	Profile contribution
Body size/passability-related self-representation	Body dimensions relative to spatial constraints. Tasks: aperture choice, aperture series, detour/pass-through, postural adjustment, near-field/aquatic/flight passability (Schiffner et al., 2014; Lenkei et al., 2020; Khvatov, 2025).	Passable vs. non-passable; first approach vs. first attempt; pre-contact refusal vs. post-contact correction; aperture-to-body ratio. Variables: latency, inspection, first attempt, passage, detour, rotation, refusal, correction, transfer.	Strong: individually scaled early choices and transfer to novel configurations. Suggestive: passable/non-passable discrimination with limited controls. Weak: largest-opening rule, post-contact correction, position/side bias, odor/tactile traces, learning, chance (Shettleworth, 2010; Dickinson, 2012; Khvatov et al., 2024).	Spatial body-constraint component. May dissociate from MSR, body-weight/support, agency, or self-signal tasks.
Body weight/support-related self-representation	Body weight, weight distribution, and body position as mechanical conditions of support, obstruction, or object movement. Tasks: body-as-obstacle, object retrieval, blanket/cart, support choice, unstable support (Dale and Plotnik, 2017; Khvatov et al., 2021a; Vanhooland et al., 2025).	Adequate vs. inadequate support; stable vs. unstable surface; body-on-object vs. body-off-object; first-trial success vs. correction. Variables: failed pulls, stepping off, repositioning, support choice, resistance/stability testing, transfer.	Strong: anticipatory repositioning, weight-appropriate support choice, transfer to novel mechanical configurations. Suggestive: correction after feedback. Weak: resistance cues, repeated failure, commands, social cueing, fear, object preference, chance stepping off (Dale and Plotnik, 2017; Lenkei et al., 2021; Vanhooland et al., 2025).	Mechanical body-support/body-obstruction component. May dissociate from passability, MSR, agency, or self-signal tasks.
Agency-related self-representation	Relation between self-produced action and sensory/environmental consequences. Tasks: action–outcome contingency, controllable/uncontrollable outcomes, delayed/distorted feedback, yoked control, tool/touchscreen/vocal feedback (Osmanski and Dooling, 2009; Kaneko and Tomonaga, 2011; Terao et al., 2026).	Controllable vs. uncontrollable; contingent vs. yoked/non-contingent; immediate vs. delayed/distorted feedback; self-generated vs. external effects. Variables: action rate, persistence, preference for control, checking, correction, abandonment, transfer.	Strong: differential behavior under contingency manipulation, correction after feedback violation, yoked/matched-reward controls, transfer. Suggestive: contingency sensitivity with limited controls. Weak: instrumental learning, reward preference, novelty, motor perseveration, accidental contingency, cueing (Heyes, 1994; Dickinson, 2012; Zentall et al., 2014).	Action-authorship/controllability component. May dissociate from appearance, passability, support, or self-signal tasks.
Visual appearance-related self-representation	Visible body image and bodily appearance. Tasks: mirror exposure, contingency checking, mirror-guided inspection, mark/sham-mark tests, video/delayed-video/photo variants (Gallup, 1970; Reiss and Marino, 2001; Plotnik et al., 2006; Prior et al., 2008).	Mirror vs. non-reflective object; visible mark vs. sham/control mark; pre-mirror vs. mirror exposure; self-directed behavior before vs. after mark detection. Variables: social-to-self-directed shift, mirror exploration, contingency checking, mark-directed action.	Strong: mirror-guided self-directed action toward a visible non-tactile/non-olfactory body change. Suggestive: contingency checking or mirror-guided inspection without mark-directed action. Weak: social response, mark irritation, tactile/olfactory cues, training, low motivation, morphology, instrumental mirror use (Gallup, 1970; Plotnik et al., 2006; de Waal, 2019).	Visual appearance component. MSR should be reported separately and may dissociate from body-size, support, agency, or self-signal modules.
Modality-specific self-signal recognition	Own individual signals relative to familiar, unfamiliar, modified-own, modified-other, or external signals. Tasks: own/other odor, modified-own signal, familiar/unfamiliar controls, playback, altered/delayed feedback, species-specific signal tasks (Bekoff, 2001; Horowitz, 2017; Freiburger et al., 2024).	Own vs. familiar/unfamiliar other; own vs. modified-own; modified-own vs. modified-other; normal vs. delayed/distorted feedback. Variables: sampling duration, approach, re-investigation, marking, orientation, vocal/social response, correction under modified feedback.	Strong: self-specific or modified-self response after novelty, familiarity, salience, contamination, habituation/dishabituation, and individual-recognition controls. Suggestive: own/other discrimination with partial controls. Weak: novelty, familiarity, intensity, territoriality, general signal discrimination (Gallup and Anderson, 2018; Akçay and Beecher, 2020).	Signal- or identity-related component, especially in non-visual species. May dissociate from MSR, passability, support, or agency tasks.

The table presents a compact version of the comparative operational matrix for constructing species-specific profiles of animal self-representation. Each row represents one candidate module and summarizes the target self-related parameter, main task families, quantifiable contrasts, key evidential logic, and contribution to a modal-modular profile. The full matrix, including species-specific access, epistemic and pragmatic actions, extended dependent variables, validation routes, supporting and weakening patterns, and expected module dissociations, is provided in Supplementary Table 1.

This operational scheme also defines boundary conditions for the model. The interpretation of a specific module is weakened when behavior is not linked to the target self-related parameter, the predicted behavioral contrast is absent, the effect disappears under control conditions, the result is fully explained by learning, sensorimotor calibration, novelty, or a simple preference rule, or transfer is absent where it is predicted. The modal-modular architecture of the MMM as a whole would be challenged if measures across all proposed modules consistently covaried as a single general factor, tasks within a module failed to show convergence, predicted dissociations were not replicated, and self-related parameters added no explanatory value beyond affordance-, calibration-, or reinforcement-based accounts.

### 4.2 Module-specific operationalization and validation

The following subsections operationalize the five candidate modules introduced in Section 3.3 in the same order: body size/passability, body weight/support, agency/action-control, visual appearance-related self-representation, and modality-specific self-signal recognition. Whereas Section 3.3 defined these modules as functional components of the MMM, the present section specifies their task families, behavioral contrasts, evidence levels, alternative explanations, control conditions, validation routes, and contribution to species-specific modal-modular profile construction.

#### 4.2.1 Body size and passability-related self-representation

The body size/passability module concerns the relation between an animal’s bodily dimensions and spatial constraints. This is one of the clearest operational domains of bodily self-representation when tasks use individually scaled parameters, early-trial performance, transfer, and controls for alternative explanations.

1. Main task families: passability can be tested through single- and multiple-aperture tasks, aperture series, manipulations of width, height, shape, or orientation, detour versus pass-through paradigms, postural-adjustment tasks, near-field, aquatic, and flight passability tasks, and shell or shelter selection tasks. These paradigms address whether the animal relates obstacle geometry to its own bodily parameters (Schiffner et al., 2014; Krieger et al., 2020; Lenkei et al., 2020; Ravi et al., 2020; Khvatov et al., 2023).
2. Quantifiable behavioral contrasts and dependent variables: key contrasts include passable versus non-passable apertures, small-passable versus large-non-passable options, first approach versus first penetration attempt, pre-contact refusal versus post-contact correction, familiar versus novel configurations, and aperture-to-body ratio effects. Dependent variables include approach latency, inspection, contacts or probes, first attempt, first-attempt success, full passage, aborted attempts, detour choice, body rotation, postural adjustment, correction, and transfer (Lenkei et al., 2020; Ravi et al., 2020; Horowitz et al., 2021; Khvatov et al., 2021b; Pongrácz et al., 2023).
3. Epistemic and pragmatic actions: epistemic actions include approach without commitment, edge inspection, head insertion, and tactile, vibrissal, proprioceptive, or hydrodynamic sampling. Pragmatic actions include first penetration attempt, full passage, detour selection, body rotation, refusal of a non-passable aperture, or selection of the passable option. This distinction helps specify how information about the body–obstacle relation may be obtained and used in action selection (Kirsh and Maglio, 1994; Khvatov, 2025).
4. Evidence levels: strong evidence is obtained when behavior scales with individual body dimensions rather than with absolute aperture properties. Especially informative patterns include pre-contact avoidance of non-passable options, first attempts directed toward a passable aperture, transfer to novel sizes, shapes, or positions, and avoidance of a simple largest-opening strategy when several options are passable (Lenkei et al., 2020; Khvatov et al., 2023; Khvatov, 2025). Suggestive evidence includes passable/non-passable differentiation under limited controls, after learning, or without demonstrated transfer. Insufficient evidence occurs when results are explained by collision-based correction, largest-opening preference, positional bias, training, odor trails, chance, or another simple rule.
5. Alternative explanations, control conditions, and falsifiability: alternative explanations include larger-opening preference, positional or side bias, odor trails, learning of a fixed aperture, post-collision avoidance, experimenter cues, fatigue, stress, uncontrolled lighting or flow, and chance. Strong designs require counterbalancing, randomized sequences, individually scaled parameters, novel configurations, separation of first approach from first attempt, odor and tactile controls, and analysis of first- or early-trial behavior (Lenkei et al., 2020; Khvatov et al., 2024; Starić et al., 2026). The module-specific interpretation would be weakened or falsified if behavior were determined by absolute aperture size, largest-opening preference, position, learning, or post-contact correction; if first attempts did not differ between passable and non-passable options; if individual body dimensions did not predict choice; or if predicted transfer were absent.
6. Validation routes, dissociations, and profile contribution: validation requires convergence across passability tasks, transfer within the same parameter domain, robustness across aperture size, shape, orientation, and position, and reproducibility across individuals and species (Lenkei et al., 2020; Khvatov et al., 2023). Dissociations are informative when passability performance does not covary with MSR, body-as-obstacle, support-selection, or agency-related paradigms (Laurenzi et al., 2025; Vanhooland et al., 2025). In a species-specific profile, this module indicates how an animal uses its own bodily dimensions to regulate movement through spatially constrained environments.

#### 4.2.2 Body weight and support-related self-representation

The body weight/support module concerns how an animal takes into account its own weight, weight distribution, and mechanical effects on a support, object, or possible action. This domain is relatively well operationalized when the animal’s body functions as a source of load, obstruction, or mechanical constraint.

1. Main task families: relevant paradigms include blanket and shopping-cart-like tasks, object-retrieval tasks in which the animal obstructs the target object, support-selection tasks, bridge or platform stability tasks, weight-bearing surface choices, object-pulling tasks, suspended-box variants, and unstable-support tasks. These paradigms test whether the animal relates object or support properties to its own body as a source of load, obstruction, or mechanical effect (Brownell et al., 2007; Dale and Plotnik, 2017; Khvatov et al., 2021a; Lenkei et al., 2021; Brosche et al., 2025; Vanhooland et al., 2025).
2. Quantifiable behavioral contrasts and dependent variables: key contrasts include adequate versus inadequate supports, stable versus unstable surfaces, obstructed versus unobstructed object-retrieval conditions, body-on-object versus body-off-object conditions, first-trial success versus correction after failure, and support-to-body-weight relations. Dependent variables include pulling or pushing while standing on the object, stepping-off latency, failed pulls, spontaneous repositioning, support choice, avoidance of unstable support, latency before commitment, first-trial success, correction after resistance feedback, and transfer to new objects or support configurations (Dale and Plotnik, 2017; Lenkei et al., 2021; Brosche et al., 2025; Vanhooland et al., 2025).
3. Epistemic and pragmatic actions: epistemic actions may include resistance testing, shifting weight, light pulling, touching or stepping on a support, probing stability, changing posture before action, or pressing a bridge with the forepaw or snout before crossing. Pragmatic actions include stepping off the obstructed object, selecting a stable or weight-appropriate support, moving the target after repositioning, crossing via the adequate support, or refusing an inadequate one. This distinction separates simple reactions to resistance from sequences in which the animal samples mechanical constraints before changing body position, selecting a support, or implementing the target action (Kirsh and Maglio, 1994; Dale and Plotnik, 2017; Khvatov et al., 2021a).
4. Evidence levels: strong evidence is obtained when the animal changes body position, selects an appropriate support, or avoids a mechanically inadequate configuration before obvious failure. Informative patterns include first-attempt success, spontaneous stepping off, selection of a weight-appropriate support, transfer to novel objects or mechanical configurations, and a distinction between testing resistance or stability and performing the target action (Dale and Plotnik, 2017; Lenkei et al., 2021; Vanhooland et al., 2025). Suggestive evidence includes correct behavior after feedback, under limited controls, or after learning. Insufficient evidence occurs when results are explained by tension, repeated failure, human commands, social cueing, fear of unstable surfaces, familiar-object preference, or chance stepping off.
5. Alternative explanations, control conditions, and falsifiability: alternative explanations include learning after failure, tension or resistance reactions, command compliance, social cueing, familiar-support preference, fear of unstable surfaces, object-attractiveness differences, chance stepping off, and reinforcement history. Strong designs require novel objects, no-command or minimally cued conditions, matched perceptual features, control supports or boxes, transfer tasks, and independent coding of failed attempts, resistance testing, and corrective actions (Dale and Plotnik, 2017; Lenkei et al., 2021; Brosche et al., 2025; Vanhooland et al., 2025). The module-specific interpretation would be weakened or falsified if behavior were determined by resistance, learning after failure, social cueing, commands, fear, or object preference; if early behavior did not differ between adequate and inadequate mechanical conditions; if body weight or body position did not predict choice; or if predicted transfer were absent.
6. Validation routes, dissociations, and profile contribution: validation requires convergence across object-retrieval, body-as-obstacle, support-selection, and unstable-support tasks, transfer within the same mechanical domain, robustness across changes in objects, supports, surfaces, and feedback conditions, and reproducibility across individuals and species (Brownell et al., 2007; Dale and Plotnik, 2017; Lenkei et al., 2021; Vanhooland et al., 2025). Dissociations are informative when weight/support performance does not covary with passability, MSR, self-signal recognition, or agency-related paradigms. In a species-specific profile, this module indicates how an animal uses its own weight, weight distribution, and body position for mechanical regulation of action.

#### 4.2.3 Agency-related self-representation: self-produced action and action control

The agency/action-control module concerns how an animal relates its own actions to their sensory or environmental consequences. This theoretically important domain requires cautious operationalization because agency-related effects may overlap with instrumental learning, reward preference, contingency sensitivity, or general action regulation.

1. Main task families: relevant paradigms include action-outcome contingency tasks, controllable versus uncontrollable stimulus tasks, delayed, distorted, or reversed feedback paradigms, self-generated versus externally generated stimulation, joystick or touchscreen control tasks, tool-mediated action tasks, yoked-control designs, and vocal feedback manipulations when the critical variable is controllability, timing, or distortion of self-produced output rather than individual acoustic recognition. These paradigms test whether an event is treated as dependent on, and controllable through, the animal’s own action (Miall et al., 1985; Osmanski and Dooling, 2009; Seed and Byrne, 2010; Kaneko and Tomonaga, 2011; Dickinson, 2012).
2. Quantifiable behavioral contrasts and dependent variables: key contrasts include controllable versus uncontrollable outcomes, self-generated versus externally generated effects, immediate versus delayed feedback, congruent versus distorted or reversed feedback, contingent versus yoked/non-contingent conditions, and intact versus disrupted action-effect relations. Dependent variables include action rate, persistence or abandonment, learning speed, preference for controllable outcomes, exploratory checking after contingency violation, disruption or correction under delayed or distorted feedback, changes in vocal or motor output, and transfer to novel actions or effectors (Miall et al., 1985; Osmanski and Dooling, 2009; Kaneko and Tomonaga, 2011; Dickinson, 2012; Pezzulo et al., 2016).
3. Epistemic and pragmatic actions: epistemic actions may include contingency checking, repeated testing, variation of amplitude or timing, exploration after feedback violation, source checking, and comparison of controllable and uncontrollable conditions. Pragmatic actions include stable use of an action to produce an outcome, controlled manipulation, tool-mediated goal achievement, vocal or motor correction, selection of a controllable option, or cessation of an ineffective action. This distinction helps specify how the animal may detect, test, and use the relation between its own action and its consequences (Kirsh and Maglio, 1994; Kaneko and Tomonaga, 2011; Pezzulo et al., 2016).
4. Evidence levels: strong evidence is obtained when the same event is processed differently depending on whether it is self-produced, externally produced, delayed, distorted, controllable, or uncontrollable. Informative patterns include behavioral change after contingency violation, preference for controllable events, correction under delayed or distorted feedback, transfer of control relations to novel conditions, and exploratory checking after disruption of the expected action-effect relation (Miall et al., 1985; Osmanski and Dooling, 2009; Kaneko and Tomonaga, 2011; Dickinson, 2012; Pezzulo et al., 2016). Suggestive evidence includes contingency sensitivity or controllability preference under limited controls. Insufficient evidence occurs when results are explained by instrumental learning, reward preference, novelty, motor perseveration, accidental action-outcome coincidence, sensory-intensity differences, experimenter cues, or low motivation.
5. Alternative explanations, control conditions, and falsifiability: alternative explanations include instrumental learning, reward preference, novelty, motor perseveration, accidental action-outcome coincidence, sensory-intensity differences, experimenter cues, and low motivation (Heyes, 1994; Dickinson, 2012; Zentall et al., 2014). Mirror, video, or vocal-feedback paradigms should be treated as agency-related only when the critical manipulation concerns action contingency, timing, distortion, or control rather than appearance, identity, or individual signal recognition (Osmanski and Dooling, 2009; Kaneko and Tomonaga, 2011; Hirata et al., 2017). Strong designs require yoked controls, non-contingent, delayed, distorted, or reversed feedback, matched reward conditions, comparison of self-generated and externally generated stimulation, and transfer to novel actions or effectors (Miall et al., 1985; Kaneko and Tomonaga, 2011; Dickinson, 2012). The module-specific interpretation would be weakened or falsified if behavior were determined by reward, learning, novelty, stimulus intensity, or accidental contingency; if controllable and uncontrollable conditions did not differ; if disruption of the action-effect relation did not elicit checking, correction, or action abandonment; or if predicted transfer were absent.
6. Validation routes, dissociations, and profile contribution: validation requires convergence across contingency, controllability, feedback-distortion, tool-use, and vocal-feedback paradigms, transfer within the domain of action-effect relations, robustness under yoked and non-contingent controls, and reproducibility across individuals and species (Miall et al., 1985; Osmanski and Dooling, 2009; Kaneko and Tomonaga, 2011; Dickinson, 2012; Pezzulo et al., 2016). Dissociations are informative when agency-related performance does not covary with MSR, passability, support-selection, or self-signal recognition paradigms (Lage et al., 2022; Laurenzi et al., 2025). In a species-specific profile, this module indicates how an animal uses the relation between its own actions and their consequences for behavioral control.

#### 4.2.4 Visual appearance-related self-representation

The appearance-related module concerns how an animal uses visual information about its own body, primarily a reflection or image, to detect, inspect, or modify bodily appearance. This domain has a well-developed experimental tradition, but its evidential strength depends on species-specific visual ecology, motivation to inspect appearance, and motor ability to act on the relevant body part.

1. Main task families: relevant paradigms include mirror exposure, mirror-guided self-inspection, contingency checking, mark-test variants, sham-mark controls, and video-, delayed-video-, or photo-based self-recognition paradigms. These tasks assess whether an animal relates a visible bodily image to its own body and uses it to guide self-directed action (Gallup, 1970, 1977; Eddy et al., 1996; Hirata et al., 2017; Kopp et al., 2021). MSR should therefore be treated as a specific paradigm for visual appearance-related self-representation rather than as a universal test of self-representation or self-awareness as a whole (Suddendorf and Butler, 2013; de Waal, 2019; Birch et al., 2020; Lage et al., 2022).
2. Quantifiable behavioral contrasts and dependent variables: key contrasts include mirror versus non-reflective object conditions, visible mark versus sham/control mark conditions, pre-mirror versus mirror exposure phases, self-directed behavior before versus after mark detection, immediate versus delayed visual feedback, and trained versus spontaneous self-directed responses. Dependent variables include reduction of social or aggressive responses, mirror exploration, contingency checking, looking behind the mirror, inspection of visually inaccessible body parts, latency, frequency and duration of self-directed behavior, mark-directed touching or removal, and responses to visible, sham, and control marks (Gallup, 1970; Plotnik et al., 2006; Prior et al., 2008; Hirata et al., 2017; Kopp et al., 2021).
3. Epistemic and pragmatic actions: epistemic actions include mirror exploration, movement–reflection contingency testing, looking behind the mirror, postural changes, inspection of visually inaccessible body parts, and exploration of unusual visual information. Pragmatic actions include mark-directed touching, mark removal, mirror-guided action toward a visually inaccessible body part, or visually guided modification of bodily appearance. This distinction separates exploration of mirror correspondence from actions in which the animal uses the reflection to act on its own body (Gallup, 1970; Plotnik et al., 2006; Hirata et al., 2017; Kopp et al., 2021).
4. Evidence levels: strong evidence is obtained when, after mirror familiarization, the animal shifts from social or aggressive responses to self-directed behavior, uses the reflection to inspect its own body, responds to a visible but non-tactile and non-olfactory mark, and directs action toward its own body rather than the mirror (Gallup, 1970; Reiss and Marino, 2001; Plotnik et al., 2006; Prior et al., 2008). Suggestive evidence includes contingency checking, mirror-guided inspection, instrumental mirror use, or intermediate mirror understanding without clear mark-directed behavior (Rochat, 2003; de Waal, 2019; Lage et al., 2022). Insufficient evidence occurs when responses are explained by social reaction to the reflection, mark irritation, tactile or olfactory cues, training, instrumental mirror use without self-recognition, or low motivation to inspect appearance.
5. Alternative explanations, control conditions, and falsifiability: alternative explanations include treating the reflection as another individual, aggression or avoidance, instrumental mirror use without self-recognition, mark irritation, tactile or olfactory cues, training effects, insufficient motivation, and morphological inability to perform the required action (Heyes, 1994; Plotnik et al., 2006; de Waal, 2019; Vanhooland et al., 2025). Strong designs require non-reflective controls, sham or invisible marks, control marks, assessment of spontaneous behavior before training, and consideration of species-specific sensory, motivational, and motor constraints (Suddendorf and Butler, 2013; de Waal, 2019; Birch et al., 2020; Lage et al., 2022). The module-specific interpretation would be weakened or falsified if behavior were determined by social responses, mark irritation, tactile or olfactory cues, training, or instrumental mirror use; if visible marks did not elicit stronger self-directed responses than sham/control marks; if action were directed toward the mirror rather than the animal’s own body; or if predicted mirror/control differences were absent.
6. Validation routes, dissociations, and profile contribution: validation requires convergence across mirror exposure, contingency checking, mark-test, mirror-guided inspection, and delayed-video paradigms, robustness under sham/control-mark conditions, reproducibility across individuals and species, and consideration of the species’ visual, motivational, and motor suitability for the task (Gallup, 1970; Reiss and Marino, 2001; Plotnik et al., 2006; Hirata et al., 2017; Kopp et al., 2021). Dissociations are informative when MSR performance does not covary with body-as-obstacle, passability, support-selection, agency-related, or self-signal recognition tasks (de Waal, 2019; Laurenzi et al., 2025; Vanhooland et al., 2025). In a species-specific profile, this module indicates how an animal uses visually mediated information about its own body to inspect or modify bodily appearance.

#### 4.2.5 Modality-specific self-signal recognition: olfactory, acoustic, and other paradigms

Modality-specific self-signal recognition concerns how animals use their own individual signals, including odor, vocalizations, acoustic, chemical, electric, vibratory, hydrodynamic, or other species-specific cues, as self-related information. This domain should be treated as a family of candidate modules because each sensory system defines distinct access conditions, behavioral responses, and interpretative criteria.

1. Main task families: relevant paradigms expose the animal to its own, familiar-other, unfamiliar-other, modified-own, modified-other, or externally generated signal. They include own-odor versus conspecific-odor tests, modified-own-odor tests, familiar versus unfamiliar odor controls, playback of own versus other vocalizations, modified-own-call or altered-feedback paradigms, delayed auditory feedback tasks, self-generated versus externally generated species-specific signals, and electric, vibratory, chemical, or hydrodynamic self-signal discrimination tasks (Bekoff, 2001; Poirier et al., 2009; Horowitz, 2017; Cazzolla Gatti et al., 2021; Szabo and Ringler, 2023; Freiburger et al., 2024). These paradigms should be modality-specific because each modality imposes its own access conditions and interpretative criteria (Stevens, 2013; Gallup and Anderson, 2018; Bräuer et al., 2020).
2. Quantifiable behavioral contrasts and dependent variables: key contrasts include own versus familiar-other signals, own versus unfamiliar-other signals, own versus modified-own signals, modified-own versus modified-other signals, familiar-signal preference versus self-referential response, normal versus delayed/distorted feedback, and self-generated versus externally generated signals. Dependent variables include sniffing, listening or inspection duration, approach latency, repeated sampling, re-investigation, avoidance or attraction, scent marking, orientation to playback, social responses, vocal replies, changes in vocalization, and behavioral disruption or correction under modified or delayed feedback (Bekoff, 2001; Osmanski and Dooling, 2009; Poirier et al., 2009; Horowitz, 2017; Szabo and Ringler, 2023; Freiburger et al., 2024).
3. Epistemic and pragmatic actions: epistemic actions include sniffing, repeated sampling, stimulus comparison, orienting to playback, inspecting a modified self-signal, source checking, and renewed investigation after signal alteration. Pragmatic actions may include scent marking, approach or avoidance, contact with the stimulus source, vocal reply, social display, vocal correction or suppression under altered feedback, or selective interaction with one signal class. Since pragmatic actions are often less sharply expressed than in passability or MSR tasks, repeated sampling, response change, and selective interaction with modified-own signals are especially informative (Osmanski and Dooling, 2009; Poirier et al., 2009; Horowitz, 2017; Freiburger et al., 2024).
4. Evidence levels: strong evidence is obtained when the animal discriminates among own, familiar-other, unfamiliar-other, modified-own, and modified-other signals, and the response to the modified-own signal remains specific after controls for novelty, familiarity, intensity, complexity, contamination, and motivational salience (Horowitz, 2017; Gallup and Anderson, 2018; Akçay and Beecher, 2020; Szabo and Ringler, 2023; Freiburger et al., 2024). Informative patterns include changes in orientation, social behavior, vocalization, or action when the expected relation between a self-signal and its altered or external form is disrupted (Osmanski and Dooling, 2009; Poirier et al., 2009). Suggestive evidence includes own/other discrimination under limited controls. Insufficient evidence occurs when results are explained by novelty, habituation/dishabituation, familiarity, individual recognition, signal intensity, stimulus complexity, territorial response, or general signal discrimination.
5. Alternative explanations, control conditions, and falsifiability: alternative explanations include novelty effects, habituation/dishabituation, familiarity, individual recognition, odor or sound intensity, stimulus complexity, contamination, territorial responses without self-recognition, reactions to unfamiliar individuals, experimenter cues, and uncontrolled motivational differences (Horowitz, 2017; Gallup and Anderson, 2018; Akçay and Beecher, 2020). Strong designs require matched control stimuli, familiar and unfamiliar controls, modified-self and modified-other conditions, counterbalanced presentation, control of stimulus intensity and contamination, repeated testing with novel stimulus combinations, and, where possible, altered- or delayed-feedback conditions (Osmanski and Dooling, 2009; Horowitz, 2017; Szabo and Ringler, 2023; Freiburger et al., 2024). The module-specific interpretation would be weakened or falsified if behavior were determined by novelty, familiarity, habituation/dishabituation, intensity, contamination, territoriality, or individual recognition; if modified-own signals did not differ from modified-other or unfamiliar signals; if own-signal responses disappeared after salience controls; or if feedback alteration had no predicted effect.
6. Validation routes, dissociations, and profile contribution: validation requires convergence across own-signal, modified-own, familiar/unfamiliar control, playback, altered-feedback, and species-specific signal paradigms, robustness under novelty and familiarity controls, transfer to new signal combinations, and reproducibility within the relevant sensory modality (Bekoff, 2001; Horowitz, 2017; Cazzolla Gatti et al., 2021; Szabo and Ringler, 2023; Freiburger et al., 2024). Dissociations are informative when self-signal performance does not covary with MSR, passability, support-selection, or agency-related paradigms (Bräuer et al., 2020; Lage et al., 2022; Laurenzi et al., 2025). In a species-specific profile, this domain indicates which individual self-signals the animal uses as self-related cues and through which sensory channels they become behaviorally relevant.

## 5 General discussion

The present article builds on gradualist, multidimensional, embodied, and ecological approaches to animal self-representation beyond the visually centered logic of mirror self-recognition (de Waal, 2019; Birch et al., 2020; Lage et al., 2022; Laurenzi et al., 2025). The contribution of the Modal-Modular Model lies in translating this general research orientation into a profile-based and operational architecture for comparative analysis. The MMM is intended to link the distinction between self-processing, self-representation, self-awareness, and self-consciousness to the analysis of specific self-related parameters, sensory modalities, ecological tasks, behavioral contrasts, evidence levels, control conditions, validation routes, and species-specific modal-modular profiles. Candidate modules are therefore defined primarily by the self-related parameter and functional problem they address, whereas task families serve as operational routes for making these parameters behaviorally observable. This architecture is intended to support comparison of the different ways in which animals take their own bodies, actions, and agency into account in tasks shaped by their morphology, sensory ecology, and behavioral organization (Lenkei et al., 2020; Vanhooland et al., 2025). In this form, the MMM provides an evidence-graded operational scaffold for relating distinct experimental paradigms to one another within a unified comparative logic.

In this sense, the MMM should be understood primarily as an operational and comparative framework for organizing evidence, specifying testable distinctions, and generating research programs, rather than as a fully explanatory theory of the mechanisms of self-representation.

This operational positioning is also consistent with recent stratified and relational approaches to human–animal difference, which support a non-binary view of consciousness and continuity. Ferreira (2026), for example, argues that evolutionary continuity in consciousness, sentience, and agency can be combined with differentiated forms of human responsibility, thereby avoiding both strong anthropocentrism and undifferentiated egalitarianism. The MMM is compatible with this broader orientation, while pursuing a narrower comparative and operational aim: to specify how animal self-representation can be investigated across species through operational dimensions and evidence-graded criteria. Broader normative, educational, and planetary-health implications of multispecies responsibility remain beyond the scope of the present analysis (Ferreira and Tukaiev, 2026).

At the same time, several limitations and risks of the proposed framework should be explicitly acknowledged.

- Hypothetical status and risk of over-fragmentation of candidate modules. The proposed modules should be understood as operationally distinguishable components of a broader regulatory architecture rather than as fully autonomous cognitive mechanisms. Their empirical independence remains a working hypothesis and should be tested through within-module convergence, transfer across related tasks, dissociation analyses, and species-specific task batteries. They are introduced to differentiate target parameters, task demands, and evidential criteria (Gallagher and Daly, 2018; Laurenzi et al., 2025). Modular differentiation therefore provides an analytical strategy for comparative research, while actual behavior, ontogenetic development, and evolutionary organization are likely to involve task-dependent coupling among several components.
- Open boundary between self-representation and affordance-based regulation. In embodied paradigms, particularly in passability, support, and body-as-obstacle tasks, self-representational interpretations require careful separation from more general sensitivity to environmental affordances. Successful performance provides relevant evidence when behavior is organized by the relation between a self-related parameter and a task constraint, rather than by the external stimulus configuration alone. This distinction should therefore be treated as an open conceptual and methodological challenge rather than as a fully resolved boundary between self-representation and affordance-based regulation. Accordingly, interpretation should be supported by analyses of behavioral sequences, transfer to novel configurations, first-trial or early-trial performance, appropriate control conditions, and the distinction between epistemic and pragmatic actions (Gibson, 1979; Lenkei et al., 2020; Khvatov, 2025).
- Alternative explanations and conservative interpretation. The MMM calls for conservative interpretation whenever simpler explanations remain sufficient. Associative learning, reinforcement history, sensorimotor calibration, ecological calibration, familiarity, habituation/dishabituation, individual recognition, signal discrimination, simple preference rules, and experimenter cueing may account for some successful behavior without requiring a self-representational interpretation (Morgan, 1894; Shettleworth, 2010; Dickinson, 2012; Gallup and Anderson, 2018). A self-representational interpretation becomes stronger when these explanations are constrained by control conditions, when performance transfers to novel configurations, when behavior is individually scaled to the animal’s body, when simple perceptual or motor rules are excluded, and when meaningful dissociations emerge among candidate modules.
- Unequal evidential strength across paradigms. MSR, olfactory self-recognition, body-as-obstacle tasks, passability paradigms, agency tasks, and metacognitive-like paradigms differ in their inferential strength, susceptibility to alternative explanations, and ability to reveal the structure of self-related behavior. In the current literature, body size/passability and body weight/support domains appear more operationally robust when appropriate controls are used, whereas agency-related and modality-specific self-signal domains require more cautious evidence-graded interpretation because they may overlap with familiarity, habituation/dishabituation, associative learning, individual recognition, or general signal discrimination. The proposed battery should therefore be used as a profile-based system for weighting evidence, rather than as a simple checklist in which all positive results have equal evidential value (Gallup and Anderson, 2018; de Waal, 2019; Vanhooland et al., 2025).
- Need for further validation. The proposed battery should be regarded as a heuristic and methodological scaffold that requires further empirical validation. Its reliability, transferability across species, sensitivity to species-specific constraints, and capacity to identify stable profiles of self-representation should be assessed through species-specific batteries, transfer tests, cross-modal manipulations, dissociation analyses, and subsequent formalization of the operational variables specified here (Birch et al., 2020; Lage et al., 2022; Laurenzi et al., 2025).

Taken together, these limitations specify the conditions under which the MMM should be used: as an evidence-graded framework for organizing testable interpretations under conservative criteria.

Overall, the MMM provides a profile-based, evidence-graded, and operational framework for the comparative study of animal self-representation. From an evolutionary perspective, the framework suggests that distinct components of self-representation can be examined as relatively autonomous lines of functional differentiation: they may have emerged, intensified, weakened, or reorganized across phylogenetic lineages depending on species-specific ecology, morphology, sensory organization, and social conditions (Tinbergen, 1963; Dukas, 2004; de Waal, 2019; Lage et al., 2022). At the same time, the framework has clear limits: it does not equate all adaptive body-environment regulation with self-representation, does not assume equal evidential strength across all candidate modules, and does not propose a universal numerical index of self-representation. Future research should validate these modal-modular profiles through species-tailored task batteries, transfer tests, cross-modal manipulations, dissociation analyses, and subsequent formal or computational modeling of the operational variables specified here. This research program may help move comparative psychology from ranking species by their proximity to human self-awareness toward a more pluralistic, testable, and ecologically grounded analysis of different forms of animal self-representation.

## Data Availability

The original contributions presented in the study are included in the article/Supplementary material, further inquiries can be directed to the corresponding author.
