# Supplementary material for: The modal-modular model of animal self-representation: a comparative and operational framework
Source: Front Psychol. 2026 Jun 25;17:1885694. doi: 10.3389/fpsyg.2026.1885694 (PMC13346195; doi:10.3389/fpsyg.2026.1885694)
Supplement: Supplementary file 1 [file Table_1.DOCX]

**Supplementary Table 1. Operational matrix for candidate modules of animal self-representation**

The matrix is designed as an evidence-graded, profile-based operational scaffold for the Modal-Modular Model (MMM). It is not a rigid diagnostic checklist and does not provide a universal numerical index of animal self-representation. Instead, it specifies module-specific self-related parameters, task families, quantifiable behavioral contrasts, evidence levels, controls, validation routes, minimal requirements for profile inclusion, and patterns that would support or weaken a self-representational interpretation.

**1. Candidate functional module**

| **Analytical dimension** | **Visual appearance-related self-representation** | **Modality-specific self-signal recognition** | **Body size/passability-related self-representation** | **Body weight/support-related self-representation** | **Agency-related self-representation** |
| --- | --- | --- | --- | --- | --- |
| **1. Candidate functional module** | A candidate module concerning the use of mirror- or image-mediated visual information about one’s own body for detecting, inspecting, or modifying bodily appearance. It should be treated as an appearance-related component rather than as a universal test of self-representation as a whole. | A candidate module concerning the processing of individual self-related signals, including odor, vocalizations, chemical cues, electric fields, vibratory cues, hydrodynamic cues, or other species-specific signals. It is relevant when such signals can be linked to self/other discrimination or modified-self detection. | A candidate module concerning the relation between the animal’s bodily dimensions and spatial constraints. It addresses whether the animal can regulate action by relating its size, shape, posture, flexibility, or bodily boundaries to apertures, gaps, passages, or obstacles. | A candidate module concerning the body as a mechanical factor in support, obstruction, load, or object manipulation. It addresses whether the animal can treat its own body weight, weight distribution, or bodily position as a condition of successful action. | A candidate module concerning the relation between self-produced actions and their sensory or environmental consequences. It addresses whether the animal can distinguish controllable from uncontrollable events and self-produced from externally produced outcomes. |

**2. Target self-related parameter**

| **Analytical dimension** | **Visual appearance-related self-representation** | **Modality-specific self-signal recognition** | **Body size/passability-related self-representation** | **Body weight/support-related self-representation** | **Agency-related self-representation** |
| --- | --- | --- | --- | --- | --- |
| **2. Target self-related parameter** | The relation between a visible body image and the animal’s own bodily appearance, including reflected or displayed body parts, visible modifications, and visually inaccessible body regions made available through a mirror or image. | The relation between the animal’s own signal and other signal classes, such as familiar-other, unfamiliar-other, modified-own, modified-other, or externally generated signals. | The relation between the animal’s own body dimensions and spatial constraints, including width, height, length, diameter, posture, shape, flexibility, and deformability. | The relation between the animal’s body weight, weight distribution, bodily position, or mechanical obstruction and the requirements of a support, object, or surface. | The relation between the animal’s own action and its outcome, including contingency, controllability, temporal correspondence, feedback distortion, and self-generated versus externally generated stimulation. |

**3. Functional problem**

| **Analytical dimension** | **Visual appearance-related self-representation** | **Modality-specific self-signal recognition** | **Body size/passability-related self-representation** | **Body weight/support-related self-representation** | **Agency-related self-representation** |
| --- | --- | --- | --- | --- | --- |
| **3. Functional problem** | Detecting whether a visually mediated body image corresponds to one’s own body and using this relation to inspect or act on one’s own appearance. | Distinguishing one’s own signal from other, familiar, unfamiliar, modified, or externally altered signals in a way that is behaviorally relevant for self/other discrimination. | Determining whether the body can pass through, fit into, or negotiate a spatial constraint before or during action. | Determining whether one’s own body blocks, loads, destabilizes, or can be repositioned to enable a goal-directed action. | Determining whether an event depends on one’s own action and whether the action-effect relation remains intact, delayed, distorted, or disrupted. |

**4. Species-specific access and ecological relevance**

| **Analytical dimension** | **Visual appearance-related self-representation** | **Modality-specific self-signal recognition** | **Body size/passability-related self-representation** | **Body weight/support-related self-representation** | **Agency-related self-representation** |
| --- | --- | --- | --- | --- | --- |
| **4. Species-specific access and ecological relevance** | Primarily visual; suitable when the species has adequate visual acuity, motivation to inspect or modify appearance, ability to act on relevant body parts, and limited social or aggressive interference in response to reflections. Ecological validity should not be assumed from mirror use alone. | Depends on the species’ sensory ecology: olfactory/chemical in many mammals, reptiles, and fish; acoustic in vocal species; electric, vibratory, or hydrodynamic in specialized taxa. Ecological relevance may involve territory, social interaction, mating, or individual recognition. | May be visual, tactile, vibrissal, proprioceptive, vestibular, hydrodynamic, or multimodal. Visually oriented species may assess passability at a distance, whereas rodents, snakes, fish, or elongated animals may rely on near-field sampling. | May involve proprioceptive, tactile, vestibular, visual, and resistance-based feedback. Relevant contexts include object retrieval, body-as-obstacle tasks, unstable supports, load-bearing surfaces, and weight-dependent route choice. | Depends on the sensory consequences of action: visual, auditory, tactile, proprioceptive, or multimodal feedback. Relevant contexts include object control, instrumental action, tool use, vocal production, play, manipulation, and avoidance. |

**5. Main task families**

| **Analytical dimension** | **Visual appearance-related self-representation** | **Modality-specific self-signal recognition** | **Body size/passability-related self-representation** | **Body weight/support-related self-representation** | **Agency-related self-representation** |
| --- | --- | --- | --- | --- | --- |
| **5. Main task families** | Mirror exposure; mirror-guided self-inspection; contingency checking; mark-test variants; sham-mark controls; video- or photo-based self-recognition; delayed-video paradigms; inspection of visually inaccessible body parts through mirror or image mediation. | Own-odor versus other-odor tasks; modified-own-signal paradigms; familiar versus unfamiliar signal controls; playback of own versus other vocalizations; altered or delayed auditory feedback; chemical, electric, vibratory, hydrodynamic, or other species-specific signal tasks. | Single-aperture passability tasks; multiple-aperture choice tasks; decreasing or increasing aperture series; aperture tasks varying width, height, shape, orientation, or area; detour versus pass-through paradigms; postural-adjustment or body-rotation tasks; near-field and transfer tasks. | Blanket and shopping-cart-like tasks; object-retrieval tasks in which the animal stands on or obstructs the target object; support-selection tasks; bridge/platform stability tasks; weight-bearing surface choice; suspended-box variants; transfer tasks with novel supports or mechanical configurations. | Action-outcome contingency tasks; controllable versus uncontrollable stimulus tasks; delayed, distorted, or reversed feedback; self-generated versus externally generated stimulation; joystick, touchscreen, or tool-mediated control; yoked-control designs; transfer to novel actions or effectors. |

**6. Quantifiable behavioral contrasts**

| **Analytical dimension** | **Visual appearance-related self-representation** | **Modality-specific self-signal recognition** | **Body size/passability-related self-representation** | **Body weight/support-related self-representation** | **Agency-related self-representation** |
| --- | --- | --- | --- | --- | --- |
| **6. Quantifiable behavioral contrasts** | Mirror versus non-reflective object; visible mark versus sham/control mark; pre-mirror versus mirror exposure; self-directed behavior before versus after mark detection; immediate versus delayed visual feedback; trained versus spontaneous self-directed response. | Own versus familiar-other signal; own versus unfamiliar-other signal; own versus modified-own signal; modified-own versus modified-other signal; normal versus delayed/distorted feedback; familiar signal preference versus self-referential response. | Passable versus non-passable apertures; small-passable versus large-non-passable options; first approach versus first penetration attempt; pre-contact refusal versus post-contact correction; familiar versus novel configurations; aperture-to-body ratio effects. | Adequate versus inadequate support; stable versus unstable surface; obstructed versus unobstructed object retrieval; first-trial success versus correction after failure; body-on-object versus body-off-object conditions; support-to-body-weight relation. | Controllable versus uncontrollable outcomes; self-generated versus externally generated effects; immediate versus delayed feedback; congruent versus distorted/reversed feedback; contingent versus yoked/non-contingent conditions; intact versus disrupted action-effect relation. |

**7. Core dependent variables**

| **Analytical dimension** | **Visual appearance-related self-representation** | **Modality-specific self-signal recognition** | **Body size/passability-related self-representation** | **Body weight/support-related self-representation** | **Agency-related self-representation** |
| --- | --- | --- | --- | --- | --- |
| **7. Core dependent variables** | Latency, duration, and frequency of mirror exploration; reduction of social responses; contingency checking; looking behind the mirror; self-directed behavior; mark-directed touching or removal; comparison of visible, sham, and control marks; delayed-video performance. | Sampling duration and frequency; approach latency; repeated investigation; avoidance or attraction; scent marking; orientation to playback; vocal replies; changes in vocalization; correction under modified feedback; selective interaction with one signal class. | First approach; approach latency; inspection duration; probe/contact counts; head insertion; first penetration attempt; success or failure of the first attempt; full passage; aborted attempts; detour choice; body rotation; refusal; correction; transfer performance. | Failed and successful pulls; stepping-off latency; spontaneous repositioning; support choice; avoidance of unstable support; latency before commitment; resistance testing; first-trial success; correction rate; transfer to new supports or objects. | Action rate under different contingency conditions; disruption under delayed or distorted feedback; correction under altered feedback; preference for controllable outcomes; exploratory behavior after contingency violation; abandonment of ineffective actions; transfer of control. |

**8. Epistemic actions**

| **Analytical dimension** | **Visual appearance-related self-representation** | **Modality-specific self-signal recognition** | **Body size/passability-related self-representation** | **Body weight/support-related self-representation** | **Agency-related self-representation** |
| --- | --- | --- | --- | --- | --- |
| **8. Epistemic actions** | Mirror exploration; looking behind or around the mirror; testing movement-reflection correspondence; changing posture in front of the mirror; orienting body parts toward the mirror; inspecting unusual visual information before acting. | Sniffing, listening, repeated sampling, orienting to playback, checking the source of a signal, re-investigating after signal alteration, comparing own, other, familiar, unfamiliar, and modified signals. | Approach without commitment; edge inspection; head insertion without full passage; tactile, vibrissal, hydrodynamic, or mechanosensory sampling; pausing before passage; repeated near-threshold approaches; exploratory contact before route selection. | Testing resistance; light pulling; shifting weight; touching, pressing, or stepping on support; probing stability; changing posture before action; inspecting whether one’s own body blocks object movement. | Contingency checking; repeated testing of the same action; varying amplitude, direction, or timing; exploring after feedback violation; comparing controllable and uncontrollable conditions; checking whether the same action still produces the expected effect. |

**9. Pragmatic actions**

| **Analytical dimension** | **Visual appearance-related self-representation** | **Modality-specific self-signal recognition** | **Body size/passability-related self-representation** | **Body weight/support-related self-representation** | **Agency-related self-representation** |
| --- | --- | --- | --- | --- | --- |
| **9. Pragmatic actions** | Directed touching or removal of the mark; mirror-guided action toward a visually inaccessible body part; grooming, inspection, or manipulation guided by the reflected image; selective self-directed action after detecting a visual change. | Scent marking; approach to or avoidance of a signal source; contact with the stimulus source; vocal reply; social display; correction or suppression of vocal output under altered feedback; selective action toward a signal class. | First penetration attempt; full passage; detour selection; body rotation; postural adjustment; selection of the passable aperture; refusal to attempt a non-passable aperture; route selection after inspection; passage through a novel configuration. | Stepping off the obstructed object; selecting an adequate support; moving the target object after repositioning; crossing via a stable support; refusing an inadequate support; changing route after stability testing; retrieving an object after removing bodily obstruction. | Controlled manipulation; stable use of an action to produce an outcome; tool-mediated goal achievement; vocal or motor correction; selection of a controllable option; cessation of an ineffective action; recalibration under altered feedback; transfer to a novel effector. |

**10. Suggestive evidence criteria**

| **Analytical dimension** | **Visual appearance-related self-representation** | **Modality-specific self-signal recognition** | **Body size/passability-related self-representation** | **Body weight/support-related self-representation** | **Agency-related self-representation** |
| --- | --- | --- | --- | --- | --- |
| **10. Suggestive evidence criteria** | Mirror exploration, contingency checking, reduction of social responses, or mirror-guided inspection may be suggestive when they differ from non-reflective controls, but remain insufficient for strong appearance-related self-representation without selective self-directed action or mark-related controls. | Differential responses to own, other, or modified-own signals are suggestive when they exceed simple familiar/unfamiliar discrimination, but novelty, intensity, and dishabituation controls are incomplete. | Differential approach, latency, hesitation, or partial adjustment to passable versus non-passable openings is suggestive when the task is body-scaled, but transfer, first-trial performance, or control of simple size preferences is limited. | Stepping off, avoiding weak supports, or correcting blocked actions is suggestive when it occurs before repeated failure, but strong interpretation requires transfer and controls against simple resistance feedback or command following. | Differential behavior under controllable versus uncontrollable or altered feedback is suggestive when it exceeds reward preference, but requires yoked/non-contingent controls and transfer for stronger interpretation. |

**11. Strong evidence criteria**

| **Analytical dimension** | **Visual appearance-related self-representation** | **Modality-specific self-signal recognition** | **Body size/passability-related self-representation** | **Body weight/support-related self-representation** | **Agency-related self-representation** |
| --- | --- | --- | --- | --- | --- |
| **11. Strong evidence criteria** | Strong evidence requires self-directed use of the reflection after familiarization, selective response to visible but not tactile/olfactory marks, action toward one’s own body rather than the mirror, spontaneous performance, and appropriate non-reflective, sham, or control-mark comparisons. | Strong evidence requires discrimination among own, other, familiar, unfamiliar, modified-own, and where possible modified-other signals, with matched controls excluding novelty, intensity, complexity, familiarity, contamination, or dishabituation. Stronger cases involve self-referential action or feedback correction. | Strong evidence requires behavior scaled to the individual’s body rather than absolute aperture size, pre-contact or early-trial selection of passable options, exclusion of the largest-opening rule, transfer to novel shapes or positions, and linkage between epistemic sampling and selective pragmatic attempts. | Strong evidence requires anticipatory repositioning, selection of weight-appropriate support, first-trial or early-trial success, transfer to novel supports or objects, and separation of resistance testing from direct target action. The animal behaves as if its body is a mechanical condition of the task. | Strong evidence requires differential processing of the same event depending on whether it is self-produced, externally produced, delayed, distorted, controllable, or uncontrollable; contingency violation should alter behavior, and performance should survive matched reward and yoked-control comparisons. |

**12. Insufficient or weak evidence**

| **Analytical dimension** | **Visual appearance-related self-representation** | **Modality-specific self-signal recognition** | **Body size/passability-related self-representation** | **Body weight/support-related self-representation** | **Agency-related self-representation** |
| --- | --- | --- | --- | --- | --- |
| **12. Insufficient or weak evidence** | Social habituation to the mirror, mirror exploration alone, looking behind the mirror, trained mark touching, or mark responses with tactile/olfactory cues uncontrolled are insufficient for strong appearance-related self-representation. | Longer investigation of modified-own signals, preference for familiar or unfamiliar signals, individual recognition, or response to stimulus intensity/complexity is insufficient when novelty, habituation/dishabituation, contamination, or familiarity are uncontrolled. | Success after extensive training, choosing the largest aperture regardless of passability, correction only after collision, absence of individual scaling, or failure to separate first approach from first attempt provides weak evidence. | Correction only after repeated failure, command-driven stepping off, preference for familiar supports, fear of unstable surfaces, no transfer, or reliance on immediate local resistance cues provides weak evidence. | Simple instrumental learning, reward preference, action-outcome association without contingency manipulation, absence of yoked/non-contingent controls, or no response to delayed/distorted feedback provides weak evidence. |

**13. Alternative explanations to control**

| **Analytical dimension** | **Visual appearance-related self-representation** | **Modality-specific self-signal recognition** | **Body size/passability-related self-representation** | **Body weight/support-related self-representation** | **Agency-related self-representation** |
| --- | --- | --- | --- | --- | --- |
| **13. Alternative explanations to control** | Response to reflection as another individual; aggression or avoidance; instrumental mirror use without self-recognition; mark irritation; tactile or olfactory cues; training effects; low motivation to touch the mark; inability to reach the marked body part. | Novelty effects; habituation or dishabituation; intensity or complexity differences; contamination; territorial or emotional responses; simple familiar/unfamiliar discrimination; individual recognition without self-reference; experimenter cues; motivational differences. | Preference for larger openings; side or position bias; odor trails; fixed-aperture learning; simple avoidance after collision; experimenter cues; fatigue or stress; uncontrolled lighting, tactile traces, or water-flow differences. | Simple learning after failure; reaction to tension or resistance; experimenter commands; social cueing; preference for familiar supports; fear of unstable surfaces; object attractiveness; chance stepping off. | Simple instrumental learning; reward preference; stimulus novelty; motor perseveration; accidental action-outcome coincidence; sensory intensity differences; experimenter cues; lack of motivation to control the outcome. |

**14. Recommended control conditions**

| **Analytical dimension** | **Visual appearance-related self-representation** | **Modality-specific self-signal recognition** | **Body size/passability-related self-representation** | **Body weight/support-related self-representation** | **Agency-related self-representation** |
| --- | --- | --- | --- | --- | --- |
| **14. Recommended control conditions** | Non-reflective object controls; sham marks; invisible or tactile-control marks; no-mark baseline; delayed-video conditions; control for prior training; matched motivation; assessment of ability to touch the relevant body part; coding before and after familiarization. | Own, familiar-other, unfamiliar-other, modified-own, and modified-other stimuli; matched intensity and complexity; contamination control; counterbalanced presentation; repeated novel combinations; playback controls; habituation/dishabituation controls. | Counterbalanced aperture positions; randomized sequences; individual scaling; novel configurations; separation of first approach and first attempt; odor/tactile trace control; first-trial or early-trial analysis; blinded coding where possible. | Novel objects and supports; no-command or minimally cued conditions; matched perceptual features; control boxes/supports; transfer tests; independent coding of failed attempts, resistance testing, stepping off, and corrective actions. | Yoked controls; non-contingent feedback; delayed, distorted, or reversed mappings; matched reward conditions; comparison of self-generated and externally generated stimulation; transfer to novel actions or effectors; control of feedback intensity. |

**15. Validation routes**

| **Analytical dimension** | **Visual appearance-related self-representation** | **Modality-specific self-signal recognition** | **Body size/passability-related self-representation** | **Body weight/support-related self-representation** | **Agency-related self-representation** |
| --- | --- | --- | --- | --- | --- |
| **15. Validation routes** | Validation should combine mark-test controls, mirror-guided inspection, delayed or altered visual feedback, replication across contexts, and comparison with non-visual modules to avoid treating MSR as a general proxy for all self-representation. | Validation should use modified-own versus modified-other comparisons, cross-signal replication, habituation/dishabituation controls, feedback manipulation, transfer to novel signal combinations, and separation of self-signal recognition from individual recognition. | Validation should use multiple aperture forms, new positions, altered posture or body configuration, individual scaling, early-trial performance, cross-task convergence, and dissociation from visual appearance or self-signal tasks. | Validation should use different support materials, novel objects, no-command variants, body-as-obstacle and support-selection tasks, transfer across mechanical configurations, and dissociation from simple resistance learning. | Validation should use yoked controls, delayed or distorted feedback, controllable versus uncontrollable outcomes, transfer to novel actions or effectors, matched reward conditions, and separation from simple reinforcement learning. |

**16. Supporting pattern for MMM**

| **Analytical dimension** | **Visual appearance-related self-representation** | **Modality-specific self-signal recognition** | **Body size/passability-related self-representation** | **Body weight/support-related self-representation** | **Agency-related self-representation** |
| --- | --- | --- | --- | --- | --- |
| **16. Supporting pattern for MMM** | Spontaneous or minimally trained self-directed use of a reflected or displayed body image under appropriate controls, especially when the animal acts on its own body rather than on the mirror or image. | Self/other or modified-self discrimination that cannot be reduced to novelty, familiarity, or stimulus complexity and that produces a self-referential behavioral adjustment or feedback correction. | Performance depends on the relation between body dimensions and environmental constraints rather than on absolute stimulus size; selective pragmatic attempts may follow broad epistemic exploration and transfer to novel configurations. | The animal anticipates or detects its own body as a mechanical condition, changes position or support choice accordingly, and transfers this relation to new mechanical contexts. | The animal detects changes in controllability or action-effect correspondence, adjusts behavior when contingency is violated, and transfers control relations beyond the trained action. |

**17. Disconfirming or weakening pattern**

| **Analytical dimension** | **Visual appearance-related self-representation** | **Modality-specific self-signal recognition** | **Body size/passability-related self-representation** | **Body weight/support-related self-representation** | **Agency-related self-representation** |
| --- | --- | --- | --- | --- | --- |
| **17. Disconfirming or weakening pattern** | Behavior is fully explained by social habituation, aggression reduction, tactile/olfactory mark cues, trained responses, or instrumental mirror use without self-directed reference to the body. | Responses follow novelty, intensity, complexity, or familiar/unfamiliar gradients rather than self/other or modified-self distinctions; no self-referential action appears under matched controls. | Animals choose the largest aperture regardless of passability, fail transfer trials, show only post-collision correction, or perform only after extensive training without individual scaling. | Success depends entirely on repeated failure, command following, familiar supports, fear responses, or local resistance cues and does not transfer to new mechanical configurations. | Behavior is fully explained by reward history, does not differ between contingent and yoked/non-contingent conditions, or remains unchanged under delayed, distorted, or disrupted feedback. |

**18. Expected module dissociations**

| **Analytical dimension** | **Visual appearance-related self-representation** | **Modality-specific self-signal recognition** | **Body size/passability-related self-representation** | **Body weight/support-related self-representation** | **Agency-related self-representation** |
| --- | --- | --- | --- | --- | --- |
| **18. Expected module dissociations** | Appearance-related performance may dissociate from body-size, body-weight, self-signal, or agency-related tasks. Failure in MSR should not be taken as absence of other self-representation modules. | Self-signal recognition may be strong in species with weak visual appearance-related performance. It may also dissociate from body-size/passability and body-weight/support tasks. | Body-size/passability performance may dissociate from MSR, self-signal recognition, and agency-related tasks. Strong passability performance does not imply reflective self-consciousness. | Body-weight/support performance may dissociate from body-size/passability, MSR, or self-signal tasks. Successful BAO/support tasks do not require visual self-recognition. | Agency-related performance may dissociate from appearance, self-signal, and body-size tasks. Action-control sensitivity does not by itself demonstrate bodily appearance or identity-related self-recognition. |

**19. Minimal requirements for inclusion in a species-specific profile**

| **Analytical dimension** | **Visual appearance-related self-representation** | **Modality-specific self-signal recognition** | **Body size/passability-related self-representation** | **Body weight/support-related self-representation** | **Agency-related self-representation** |
| --- | --- | --- | --- | --- | --- |
| **19. Minimal requirements for inclusion in a species-specific profile** | The module should be included only when visual self-related information is task-relevant for the species, mirror- or image-mediated access has been established, behavior is compared with appropriate non-reflective, sham, or control conditions, and the response goes beyond social reaction, mirror exploration alone, or trained object use. | The module should be included only when the relevant signal classes are explicitly defined (e.g., own, familiar-other, unfamiliar-other, modified-own, modified-other), stimulus intensity, novelty, familiarity, and contamination are controlled, and the behavioral pattern supports self/other or modified-self discrimination rather than simple investigation or dishabituation. | The module should be included only when the animal’s relevant body dimensions are measured or otherwise specified, behavior is scaled to the body-to-constraint relation rather than absolute aperture size, and early-trial, first-choice, or transfer data weaken simple largest-opening, side-bias, or collision-learning explanations. | The module should be included only when the relation among body weight, bodily position, support, obstruction, or object movement is experimentally specified, anticipatory repositioning or support selection is coded separately from correction after failure, and command following, resistance feedback, support familiarity, or social cueing are controlled. | The module should be included only when action-effect contingency, controllability, feedback delay/distortion, or self-generated versus externally generated outcomes are experimentally manipulated, and the behavioral pattern survives matched reward, yoked, or non-contingent control comparisons where possible. |

**20. Contribution to species-specific modal-modular profile**

| **Analytical dimension** | **Visual appearance-related self-representation** | **Modality-specific self-signal recognition** | **Body size/passability-related self-representation** | **Body weight/support-related self-representation** | **Agency-related self-representation** |
| --- | --- | --- | --- | --- | --- |
| **20. Contribution to species-specific modal-modular profile** | Contributes evidence for an appearance-related component of the profile. This component should be reported separately from other modules and should not be used as a single criterion for ranking species by self-awareness. | Contributes evidence for a signal- or identity-related component only when self-referential controls are met. It is especially important for non-visual species but should be separated from individual recognition. | Contributes evidence for a spatial body-constraint component of the profile. It is central for locomotor, shelter-seeking, burrowing, flying, aquatic, or obstacle-negotiating species. | Contributes evidence for a mechanical body-support or body-obstruction component. It is especially relevant for species facing support choice, load-bearing, object retrieval, climbing, or body-as-obstacle problems. | Contributes evidence for an action-authorship or controllability component when contingency-specific controls are satisfied. It should be integrated with, but not collapsed into, body- or identity-related modules. |
